# Elevated FOXG1 and SOX2 in glioblastoma enforces neural stem cell identity through transcriptional control of cell cycle and epigenetic regulators

**DOI:** 10.1101/gad.293027.116

**Published:** 2017-04-15

**Authors:** Harry Bulstrode, Ewan Johnstone, Maria Angeles Marques-Torrejon, Kirsty M. Ferguson, Raul Bardini Bressan, Carla Blin, Vivien Grant, Sabine Gogolok, Ester Gangoso, Sladjana Gagrica, Christine Ender, Vassiliki Fotaki, Duncan Sproul, Paul Bertone, Steven M. Pollard

**Affiliations:** 1 Medical Research Council (MRC) Centre for Regenerative Medicine, University of Edinburgh, Edinburgh EH16 4UU, United Kingdom;; 2 Edinburgh Cancer Research UK Cancer Centre, University of Edinburgh, Edinburgh EH16 4UU, United Kingdom;; 3 Wellcome Trust-MRC Stem Cell Institute, University of Cambridge, Cambridge CB2 1QR, United Kingdom;; 4 Department of Cancer Biology, UCL Cancer Institute, University College London, London WC1E 6BT, United Kingdom;; 5 Centre for Integrative Physiology, University of Edinburgh, Edinburgh EH8 9XD, United Kingdom;; 6 MRC Human Genetics Unit, MRC Institute of Genetics and Molecular Medicine, University of Edinburgh, Edinburgh EH4 2XU, United Kingdom;; 7 Edinburgh Cancer Research Centre, MRC Institute of Genetics and Molecular Medicine, University of Edinburgh, Edinburgh EH4 2XU, United Kingdom

**Keywords:** glioblastoma, cell cycle, epigenetics, dedifferentiation, neural stem cell, astrocyte

## Abstract

Glioblastoma multiforme (GBM) is an aggressive brain tumor driven by cells with hallmarks of neural stem (NS) cells. GBM stem cells frequently express high levels of the transcription factors FOXG1 and SOX2. Here we show that increased expression of these factors restricts astrocyte differentiation and can trigger dedifferentiation to a proliferative NS cell state. Transcriptional targets include cell cycle and epigenetic regulators (e.g., *Foxo3*, *Plk1*, *Mycn*, *Dnmt1*, *Dnmt3b*, and *Tet3*). *Foxo3* is a critical repressed downstream effector that is controlled via a conserved FOXG1/SOX2-bound *cis*-regulatory element. *Foxo3* loss, combined with exposure to the DNA methylation inhibitor 5-azacytidine, enforces astrocyte dedifferentiation. DNA methylation profiling in differentiating astrocytes identifies changes at multiple polycomb targets, including the promoter of *Foxo3*. In patient-derived GBM stem cells, CRISPR/Cas9 deletion of *FOXG1* does not impact proliferation in vitro; however, upon transplantation in vivo, *FOXG1*-null cells display increased astrocyte differentiation and up-regulate FOXO3. In contrast, SOX2 ablation attenuates proliferation, and mutant cells cannot be expanded in vitro. Thus, FOXG1 and SOX2 operate in complementary but distinct roles to fuel unconstrained self-renewal in GBM stem cells via transcriptional control of core cell cycle and epigenetic regulators.

## Abstract (precis)

Bulstrode et al. show that increased expression of FOXG1 and SOX2 restricts astrocyte differentiation and can trigger dedifferentiation to a proliferative neural stem cell state. FOXG1 and SOX2 operate in distinct but complementary roles to fuel unconstrained self-renewal in glioblastoma stem cells via transcriptional control of core cell cycle regulators and epigenetic targets.

Glioblastoma multiforme (GBM) is a highly aggressive brain tumor driven by neural stem (NS) cell-like cells. It is increasingly clear that the transcriptional and epigenetic mechanisms that control the initiation and maintenance of NS and progenitor cells are hijacked and deregulated in GBMs (Singh et al. 2003; Patel et al. 2014; Suvà et al. 2014). Neurodevelopmental transcription factors (TFs; e.g., basic helix–loop–helix [bHLH], SOX, FOX, and HOX families) are known to be critical regulators of NS cell self-renewal and differentiation. However, TFs are difficult to “drug” with small molecules. Improved understanding of the role of these master regulators and their key downstream effectors is needed.

We reported previously that *FOXG1* is one of the most consistently overexpressed genes when comparing primary cultures of GBM-derived NS (GNS) cells and genetically normal NS cells (Engström et al. 2012). FoxG1 is a member of the forkhead box family of TFs. During development, it has an essential role in regulating forebrain radial glia/neural progenitor cell proliferation and limiting premature differentiation (Xuan et al. 1995; Martynoga et al. 2005; Mencarelli et al. 2010).

Although *FOXG1* is not genetically amplified in glioma, *FOXG1* mRNA levels in primary tumors are inversely correlated with patient survival (Verginelli et al. 2013). Recently, Liu et al. (2015) demonstrated that the oncogenic EGFR truncation (EGFRvIII)—found in a significant proportion of “classical” subtype GBMs—operates in part by triggering expression of *FOXG1*. FOXG1 protein has been shown previously to operate by attenuating the cytostatic effects of TGF-β signaling by binding and sequestration of FOXO/SMAD complexes in established glioblastoma cell lines (Seoane et al. 2004). These findings suggest that increased levels of FOXG1 in GBM might be functionally important in driving tumor growth. Evidence in favor of this hypothesis has been provided by shRNA knockdown of FOXG1 in GBM stem cells, which leads to reduced proliferation of the resulting tumors (Verginelli et al. 2013). Despite these observations, we have a poor understanding of the functional consequences of its increased levels and the downstream transcriptional targets in both NS cells and GBM stem cells.

SOX2 is an established stem cell “master” regulator highly expressed in multiple tissue stem cells, including various types of NS and progenitor cells (Arnold et al. 2011). It has important functions within the pluripotent epiblast, embryonic stem cell cultures, neuroepithelial progenitors, and multipotent radial glia (fetal, postnatal, and adult) (Avilion et al. 2003). In *Xenopus*, chicken, and mouse embryos, the constitutive expression of *Sox2* respecifies gastrulation stage progenitor cells into neuroectoderm at the expense of other lineages (Kishi et al. 2000; Zhao et al. 2004). It is genetically amplified in ∼4% of GBM samples (Brennan et al. 2013). Knockdown experiments have indicated that SOX2 is required to sustain the aggressive growth and infiltrative behavior of GBMs (Gangemi et al. 2009; Alonso et al. 2011).

Together, these studies point to an important role for FOXG1 and SOX2 in NS cells and their potential deregulation in GBM. FoxG1 and Sox2 are also established reprogramming factors: Forced coexpression can trigger direct reprogramming of fibroblasts to an NS cell-like state (Lujan et al. 2012). The excessive levels or activity of these factors in GBM may therefore operate intrinsically to restrict tumor cell differentiation through perpetual reprogramming to a radial glia-like NS cell state. Despite the frequent expression of FOXG1/SOX2 in GBM, we have only a poor understanding of their downstream transcriptional targets and how they operate to drive proliferation and limit terminal differentiation.

Here we define genome-wide transcriptional targets of both factors and show that FOXG1/SOX2 can act at shared target loci encoding core cell cycle and epigenetic regulators. Loss-of-function studies suggest that they have context-specific functions, with SOX2 essential for proliferation, while FOXG1 protects cells from differentiation cues both in vitro and in vivo. These two transcriptional regulators therefore cooperate in functionally distinct but complementary roles to limit astrocyte differentiation commitment in GBM and enforce the proliferative NS cell-like phenotype.

## Results

### Human GBM stem cells express elevated levels of FOXG1 and exhibit an open chromatin profile enriched for FOX/SOX motifs

**Figure 1.**
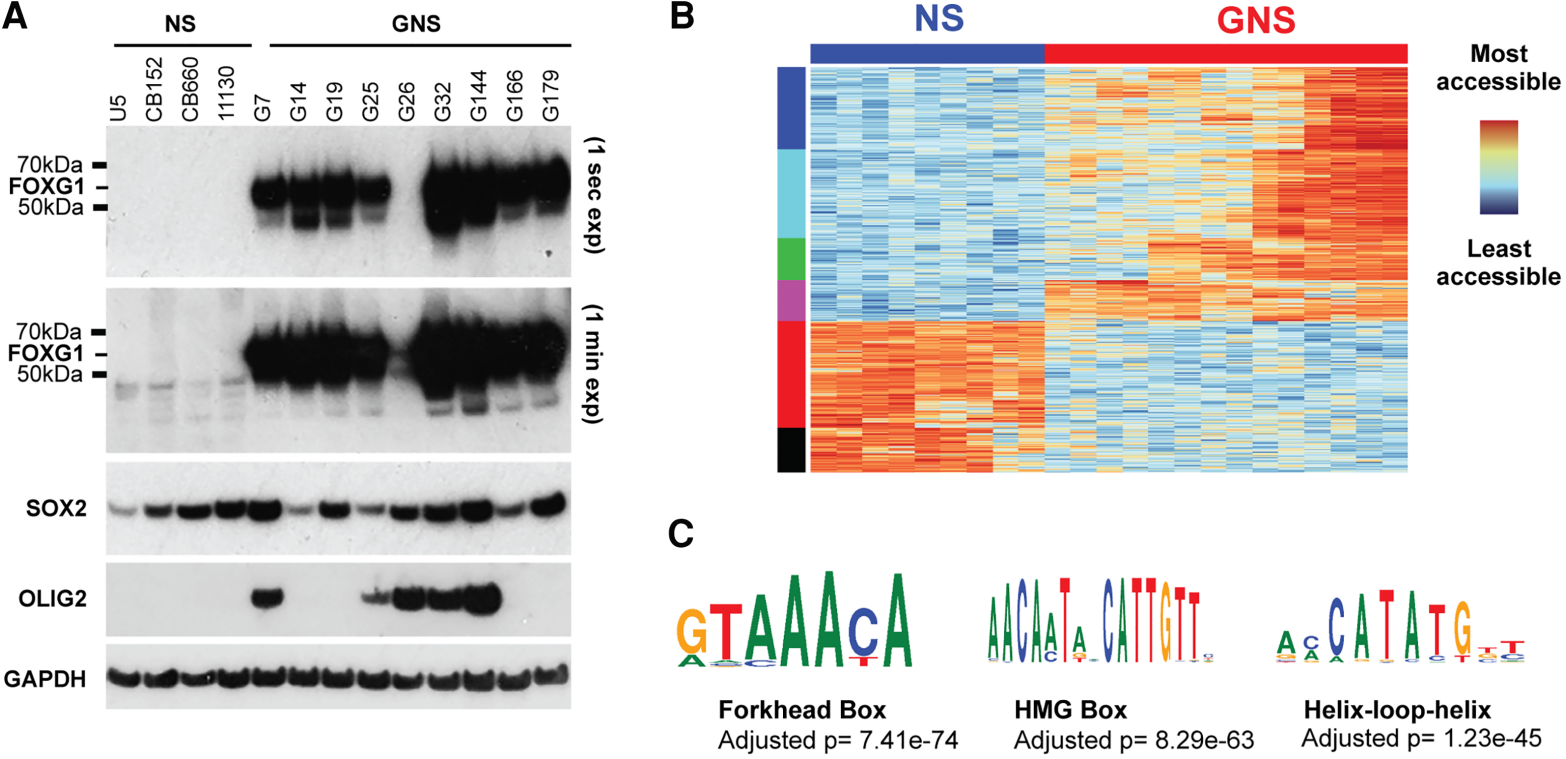
FOXG1 and SOX2 are consistently expressed at high levels across GNS cells. (*A*) Western blot to determine levels of FOXG1, SOX2, and OLIG2 expression across a set of GNS cells and normal NS controls. (*B*) ATAC-seq (assay for transposase-accessible chromatin [ATAC] using sequencing) libraries were generated in NS and GNS cells. The 100 most differentially accessible sites across biological replicates of nine GNS cell lines and four NS cells were identified and are shown in a heat map. (*C*) The most differentially accessible loci are enriched for key NS-specific TF motifs, most significantly the forkhead box motif.

To explore the role of FOXG1, we first extended our previous finding of elevated *FOXG1* mRNA expression in GBM by assessing the levels of FOXG1 protein. FOXG1 protein is consistently and highly expressed across a set of nine independent patient-derived GNS cell lines when compared with NS cells (Fig. 1A). It is also increased in a mouse glioma-initiating cell line (Supplemental Fig. S1A). SOX2 protein levels are high in both NS and GNS cells. OLIG2, a developmental TF often expressed in GBM, is more variably expressed between GNS lines (Fig. 1A).

High levels of FOXG1 in GNS cells might contribute to a modified chromatin landscape compared with karyotypically normal NS cells. To assess chromatin accessibility genome-wide in GNS and NS cells, we performed ATAC-seq (assay for transposase-accessible chromatin [ATAC] using sequencing) (Buenrostro et al. 2013). Seven independent human GNS lines (G7, G19, G25, G26, G144, G166, and G179) and four human NS cell controls were assayed in biological duplicates under proliferative culture conditions. Unsupervised clustering using the most variable sites across these libraries clearly separated GNS cells from NS cells (Fig. 1B). As expected, given patient heterogeneity, GNS cells had a greater diversity of chromatin profiles than NS cells. Interestingly, the regions identified as more accessible in GNS cells versus NS cells were enriched in the forkhead box motif and HMG box motif, which are bound by FOX and SOX factors, respectively (Fig. 1C). These data suggest that increased FOXG1 protein levels and FOX/SOX-enriched chromatin accessibility sites are a hallmark that distinguishes GNS cells from genetically normal NS cells.

### Loss of FOXG1 sensitizes NS cells to astrocyte differentiation cues

**Figure 2.**
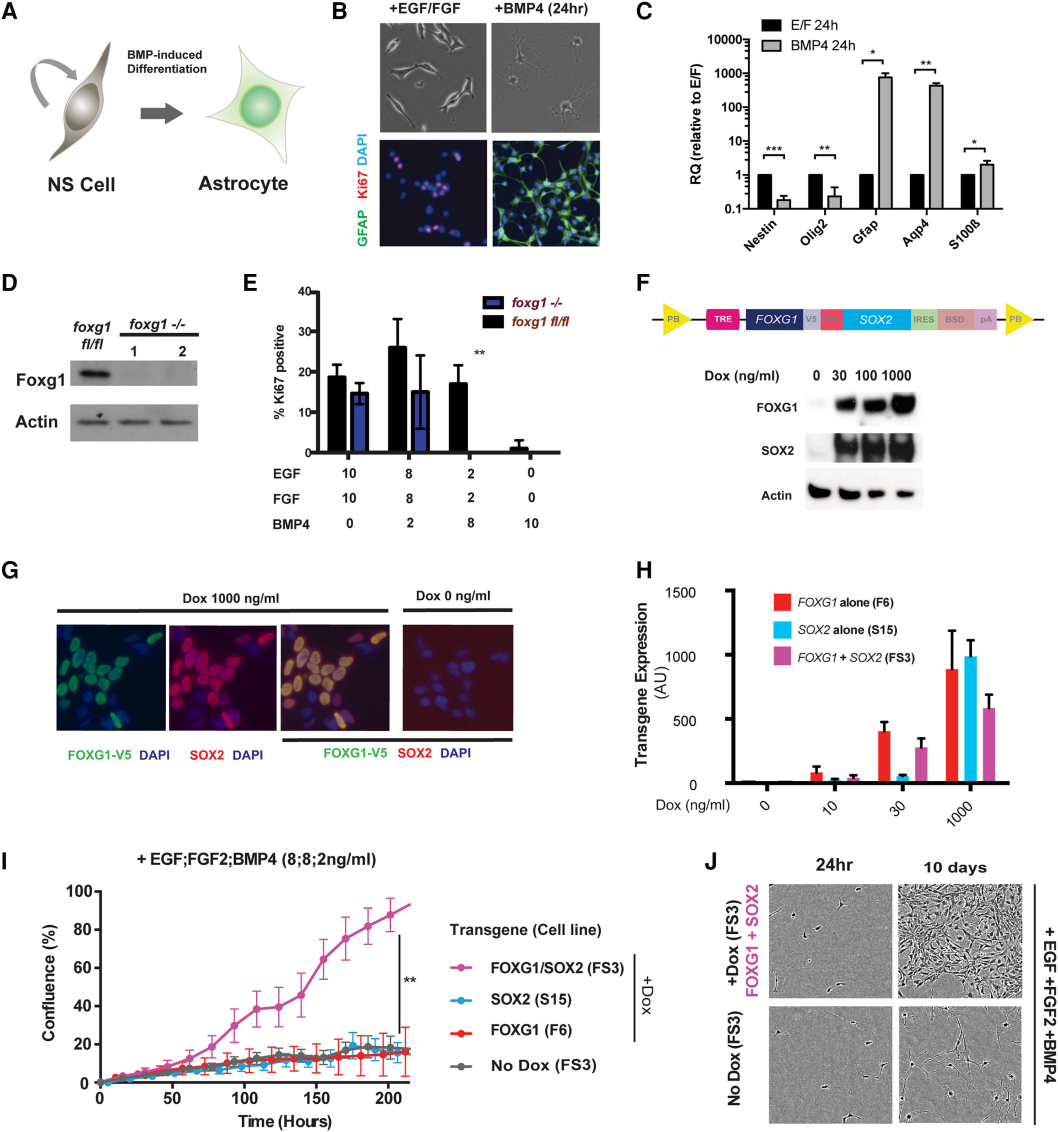
FOXG1/SOX2 overexpression can inhibit BMP-induced astrocyte differentiation. (*A*) Mouse NS cell lines provide an experimentally tractable model to study astrocyte differentiation. BMP4 treatment for 24 h is sufficient to trigger efficient differentiation: cell cycle exit, adoption of astrocyte morphological features (flattened or star-shaped), and up-regulation of Gfap. (*B*) Twenty-four hours after replacing EGF/FGF-2 with BMP4, morphological changes are accompanied by down-regulation of Ki67 and up-regulation of Gfap. (*C*) Quantitative RT–PCR (qRT–PCR) analysis shows that, at a population level, BMP4 treatment of NS cells at low density (10 cells per square millimeter) results in significant down-regulation of Nestin and Olig2 and up-regulation of astrocyte markers Gfap, Aqp4, and S100β. Mean ± SD. *n* = 3. Significance was assessed by Student's *t*-test with Holm-Sidak correction for multiple comparisons. (*) *P* ≤ 0.05; (**) *P* ≤ 0.01; (***) *P* ≤ 0.001. (*D*) Western blot to show that Foxg1 levels in clones picked following Cre treatment of Foxg1^fl/fl^ NS cells demonstrate an absence of protein expression. (*E*) Ki67 immunocytochemistry (ICC) was used to score proliferation in Foxg1 ablated cells (nanograms per milliliter). (*F*) A doxycycline (Dox)-inducible transgene cassette was designed to enable inducible coexpression of FOXG1 and SOX2. (TRE) TET-responsive element; (V5) V5 epitope tag; (P2A) porcine teschovirus-1 2A self-cleaving peptide sequence; (PB) piggyBac; (BSD) blasticidin resistance; (IRES) internal ribosome entry site. Western blot (*below*) confirmed dose-dependent increases in FOXG1 and SOX2 protein levels. (*G*) ICC for V5 and SOX2 confirms a Dox-induced (1000 ng/mL) increase in V5-FOXG1 and SOX2 levels. (*H*) Clonal lines (F6, F11, and FS3) harboring the inducible cassettes (shown in *F*) (Supplemental Fig. S2E,F) were generated, and transgene mRNA levels were determined by qRT–PCR following exposure to growth medium supplemented with different concentrations of Dox. (*I*) Growth curves for mouse NS cells cultured in medium supplemented with 8 ng/mL each mitogens EGF/FGF-2 plus 2 ng/mL BMP4 either with or without induction of FOXG1/SOX2 overexpression by Dox. Significance was assessed by Student's *t*-test: FS3 +Dox versus FS3 −Dox, *n* = 3; *P* < 0.001 at all time points after 178 h. (*J*) Phase contrast images of FS3 cells cultured in medium supplemented with 8 ng/mL each mitogens EGF/FGF-2 plus 2 ng/mL BMP4 with or without Dox supplementation after 24 h and 10 d.

Mouse NS cell cultures are a genetically and experimentally tractable model for interrogating self-renewal and differentiation commitment. Replacement of the growth factors EGF/FGF-2 with BMP4 results in prompt and uniform cell cycle exit and up-regulation of astrocyte markers, including Gfap and Aqp4 (Fig. 2A–C; Conti et al. 2005). We used this culture system to explore the specific and shared functions of Foxg1 and Sox2.

Sox2 has been shown previously to be essential for NS cell self-renewal in vitro (Gómez-López et al. 2011). To test whether Foxg1 is required for in vitro self-renewal of NS cells, we derived a new NS cell line (termed FF) from the subventricular zone (SVZ) of a previously reported adult *Foxg1*^*flox/flox*^ mouse (Supplemental Fig. S2A; Miyoshi and Fishell 2012). Transient transfection with a Cre expression plasmid resulted in biallelic excision of the *Foxg1*-coding locus. Monitoring of the *Foxg1* ablated cells over many passages using a GFP reporter of Cre excision suggested that there was no proliferation deficit (Supplemental Fig. S2B). Indeed, we could readily establish clonal *Foxg1* ablated NS cell lines (Fig. 2D). The mutant cells demonstrated no difference in proliferation or marker expression when grown in EGF/FGF-2; they also retained astrocyte differentiation potential (Supplemental Fig. S2B,C). However, in response to a combination of BMP4 and reduced amounts of EGF/FGF-2, *Foxg1*^−/−^ cells showed an increased propensity to exit cycle and differentiate (Fig. 2E). These data suggest that Foxg1 is dispensable for the maintenance of continued NS cell proliferation in vitro. It may be required instead to protect cells from differentiation commitment.

### Overexpression of FOXG1 and SOX2 in adult NS cells suppresses BMP-induced astrocyte differentiation

The high levels of FOXG1 and SOX2 in GBM stem cells may underlie the failure of differentiation commitment and unconstrained self-renewal associated with these malignancies (Carén et al. 2015). To test the consequences of increased FOXG1 and SOX2, we transfected genetically normal adult subependymal zone (SEZ)-derived mouse NS cell cultures (ANS4) with a stably integrating PiggyBac transposon plasmid carrying a tetracycline-inducible *FOXG1-2A–SOX2* expression cassette (Fig. 2F). Clonal NS cell lines were generated that responded to doxycycline (Dox) treatment by increasing expression of FOXG1 and SOX2 mRNAs in a dose-dependent manner (Fig. 2F–H). We used the human FOXG1- and SOX2-coding sequence, as the major goal was to uncover their roles in human GBM and these are each ∼97% identical to their mouse orthologs at the protein level, with 100% homology in the DNA-binding domains (Supplemental Fig. S2D). In parallel, we established inducible lines expressing FOXG1 or SOX2 individually (termed F6 and S15, respectively) (Supplemental Fig. S2E,F). FOXG1 was expressed as a fusion protein with a V5 epitope tag that enabled monitoring of transgene expression.

We cultured FS3, F6, and S15 cells in self-renewal medium (EGF/FGF-2) plus BMP4 with or without Dox. Under these conditions, parental ANS4 cells adopt an astrocyte morphology and stop proliferating. Dox-induced expression of either FOXG1 or SOX2 alone had little effect on astrocyte differentiation, and cells did not proliferate. However, coexpression of both factors restricted the differentiation response, and cultures remained proliferative (Fig. 2I,J). These data indicate that overexpression of FOXG1 and SOX2 in combination can attenuate the cytostatic effects of BMP-induced astrocyte differentiation.

### Overexpression of FOXG1 and SOX2 in post-mitotic astrocytes triggers dedifferentiation to a proliferative NS cell-like state

**Figure 3.**
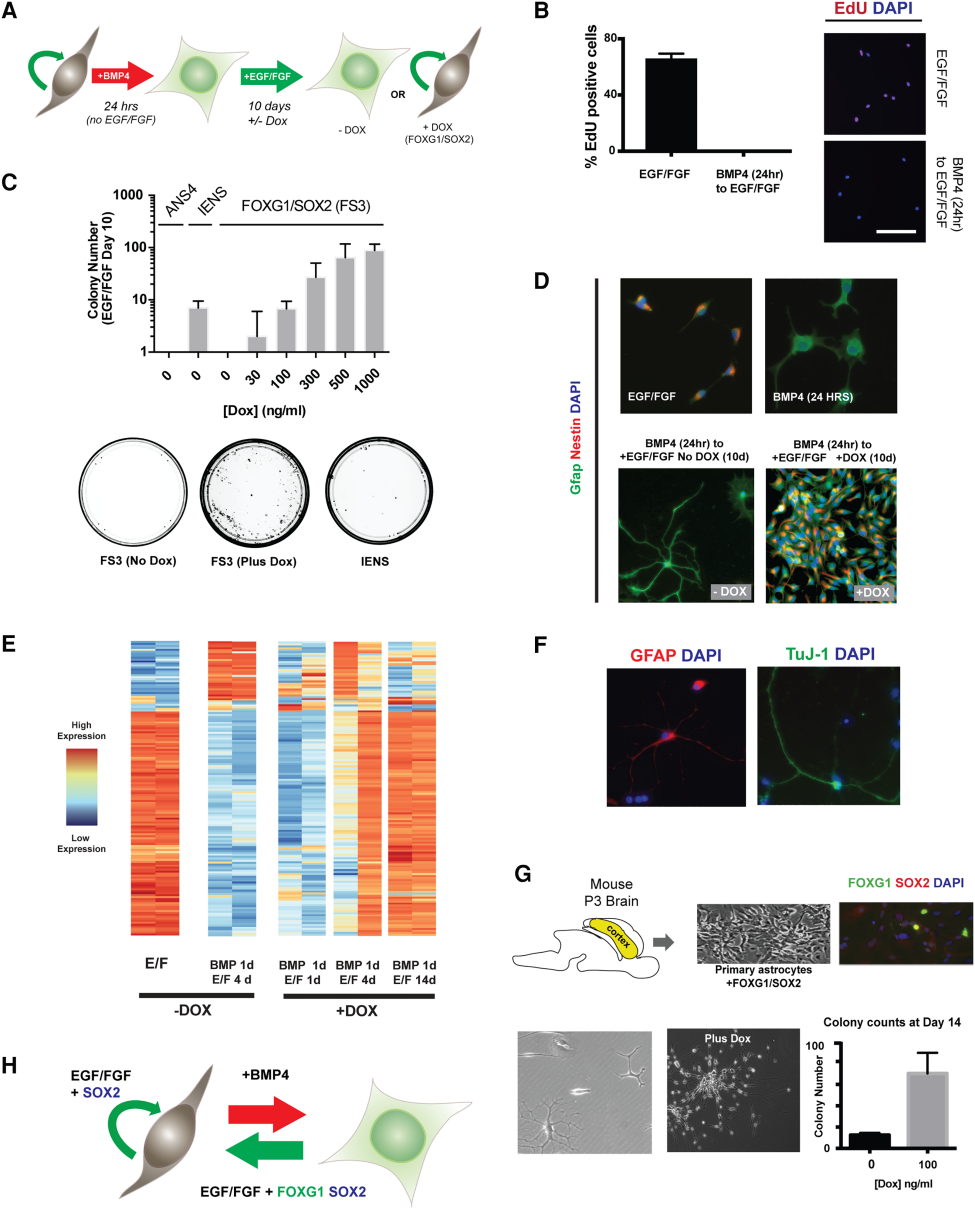
FOXG1/SOX2 drives reacquisition of NS cell identity in post-mitotic astrocytes. (*A*) Schematic of the experimental strategy used to test dedifferentiation. Cells at clonal density (10 cells per square millimeter) were treated with 10 ng/mL BMP4 for 24 h and then switched to EGF/FGF-2 medium with or without transgene induction by Dox treatment. (*B*) EdU staining shows that no rapidly cycling cells remain after 24 h of BMP4 treatment. Twenty-four hours after plating in EGF/FGF-2 or BMP4, a 24-h pulse of EdU was administered in medium containing EGF/FGF-2. Representative images of EdU staining and quantification of the percentage of EdU-positive cells are shown for each condition. Mean ± SD. *n* = 2 independent experiments. Bar, 100 µm. (*C*) Transgene dose determines the extent of colony formation after 10 d in EGF/FGF-2. *n* = 3 independent experiments. Tumor-initiating IENS cells retained colony-forming ability after BMP treatment and served as a positive control, while ANS4 cells served as a negative control. *Below* are shown example 10-cm dishes for FS3 (no Dox), FS3 plus 1000 ng/mL Dox, and IENS treated with BMP4 for 24 h and returned to EGF/FGF-2 for 10 d. FS3 cells form colonies efficiently on transgene induction. (*D*) ICC for FS3 cells showing Gfap and Nestin protein levels after 24 h in EGF/FGF-2, 24 h in BMP4, return to EGF/FGF-2 for 10 d without Dox, and return to EGF/FGF-2 for 10 d with Dox. (*E*) Heat map of the most differentially expressed transcripts across RNA sequencing (RNA-seq) libraries at various time points during dedifferentiation; biological replicates are shown for each condition, with variability at early stages due to the low absolute numbers of cells that dedifferentiate. (*F*) FS3 cells retain astrocytic and neuronal differentiation potential after long-term expansion (∼30 d), as shown by ICC for Gfap and Tuj1. (*G*) Mouse primary astrocytes were derived from a postnatal day 3 (P3) mouse cortex, and the FOXG1/SOX2-inducible transgene was introduced by lipofection. Following the described colony-forming assay, colonies were scored 2 wk following restoration of EGF/FGF-2. (*H*) A working model: In the presence of mitogens, FOXG1/SOX2 acts to restrict differentiation commitment and drive proliferation.

We next explored the functional consequences of forced expression of FOXG1 and SOX2 in differentiating astrocytes. A quantitative in vitro colony-forming assay was developed to determine whether these factors can trigger cells to re-enter cell cycle and dedifferentiate to the proliferative NS cell state (Fig. 3A). As a positive control, we used a previously reported glioma-initiating mouse NS cell line, IENS (*Ink4a/ARF* deletion, EGFRvIII overexpression) (Bachoo et al. 2002; Bruggeman et al. 2007). IENS cells express FOXG1 at high levels relative to normal NS cells (ANS4) and are highly malignant on transplantation (Supplemental Fig. S1B).

When ANS4 cells are plated at low density (10 cells per square millimeter) and cultured for 24 h in the presence of BMP4 but without the growth factors EGF/FGF-2, all cells undergo astrocyte differentiation and are subsequently unable to re-enter cell cycle when re-exposed to self-renewal medium, as assessed by EdU incorporation; i.e., they are post-mitotic and growth factor-unresponsive (Fig. 3B; Supplemental Fig. S3A). When returned to self-renewal conditions, glioma-initiating IENS cells form scattered proliferating NS cell-like colonies, consistent with a suppression of BMP-induced differentiation (Fig. 3C).

Dox-induced expression of exogenous FOXG1 and SOX2 in the growth factor-unresponsive and post-mitotic astrocytes (BMP-treated FS3 cells) resulted in dose-dependent colony formation (Fig. 3C), whereas the “no Dox”-treated controls failed to form colonies. The colonies that emerged in Dox-treated plates were rapidly cycling and comprised Nestin-high, Gfap-low cells with a characteristic NS cell morphology (Fig. 3D). FOXG1/SOX2-induced colonies were typically similar in size to control NS cell colonies (data not shown). Inspection of time-lapse imaging of dedifferentiation revealed doubling times of ∼24 h, which is comparable with parental NS cells and suggests that cells rapidly adopt a highly proliferative NS cell-like phenotype (Supplemental Fig. S3B; Supplemental Movie 1). Transcriptome profiling of these cells by RNA sequencing (RNA-seq) identified expression changes compatible with dedifferentiation and reacquisition of many features of the untreated parental cells grown in EGF/FGF-2 (Fig. 3E), such as differentiation potential (Fig. 3F). The dedifferentiated cells continued to divide upon Dox withdrawal and could be serially passaged; they exhibited morphology, proliferation, and marker expression similar to the parental FS3 cells (Supplemental Fig. S3C–E). They also remained BMP4-responsive and activated Gfap (Supplemental Fig. S3F).

To exclude the possibility that FOXG1/SOX2-induced astrocyte dedifferentiation was limited to in vitro generated astrocytes, we next introduced the *TET*–*FOXG1-2A–SOX2* transgene into freshly isolated mouse astrocytes (Fig. 3G). Induction of FOXG1 and SOX2 in primary astrocytes contributed to a significant increase in NS cell-like colonies when cells were transferred into self-renewal medium. We conclude that overexpression of FOXG1 and SOX2 in astrocytes reverses differentiation and is sufficient to drive cells to enter cell cycle and acquire a proliferative NS cell identity (Fig. 3H).

### ChIP-seq (chromatin immunoprecipitation [ChIP] combined with high-throughput sequencing) identifies FOXG1 binding at core cell cycle and methyltransferase target genes

**Figure 4.**
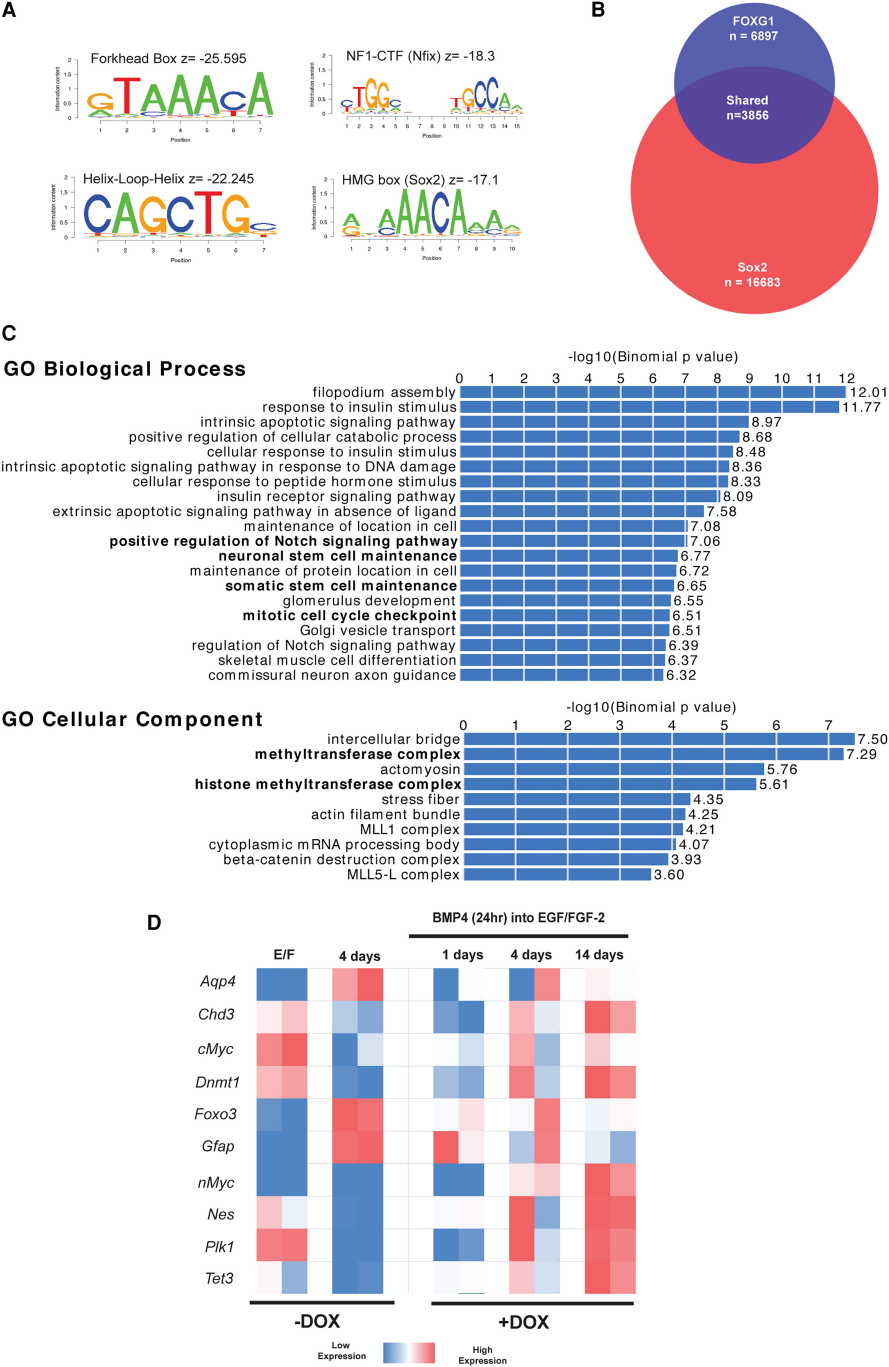
ChIP-seq of FOXG1 targets in mouse NS cells. (*A*) FOXG1-V5 ChIP-seq identifies 6897 binding peaks conserved across two separately derived mouse NS cell lines (Foxg1 ChIP mm10.bed). Motif analysis within the ChIP-seq peak regions for FOXG1-V5 reveals enrichment for the forkhead box motif as well as HLH, NF1–CTF, and HMG-box motifs. (*B*) There is extensive overlap between FOXG1- and Sox2-bound regions, with 3856 of 6897 FOXG1-bound regions also exhibiting Sox2 binding. (*C*) Shared bound regions were assigned to gene loci using the Stanford University genomic regions of enrichment annotations tool (GREAT; FOXG1_Sox2 intersect gene associations.txt) and were found to be enriched for the GO terms shown (FOXG1_Sox2 intersect gene ontology.tsv). (*D*) RNA-seq demonstrates that *Foxo3* is up-regulated after BMP4 treatment, along with astrocyte markers *Gfap* and *Aqp4*; in contrast, *Nestin* and epigenetic remodeling machinery *Tet3* and *Dnmt1* are down-regulated. NS cell expression patterns return by day 14 (+Dox).

The in vitro dedifferentiation assay provided a tractable system to define transcriptional target genes through which FOXG1 and SOX2 operate. Sox2 target genes in mouse neural progenitor cells have been defined previously using ChIP-seq (Lodato et al. 2013). Identification of FOXG1 targets has been hindered by the limitations of available native antibodies. To overcome this, we performed ChIP-seq in NS cells constitutively expressing the V5 epitope-tagged version of FOXG1, which remained functional in our earlier dedifferentiation assays (Fig. 3). Two independent NS cell lines constitutively overexpressing FOXG1-V5 were generated from either ANS4 or an independent primary adult SVZ-derived NS cell line. From the V5 ChIP-seq, we identified 6897 high-confidence binding sites shared between these cell lines, and motif enrichment analysis confirmed the canonical forkhead motif to be most significantly enriched (Fig. 4A). We also found many other neurodevelopmental lineage-affiliated TF motifs enriched at these sites, including bHLH, HMG box (the SOX family-binding motif), and CTF/NF1 factors (Fig. 4A). These are bound by TFs recognized as key components of the core circuit of self-renewal in NS cells (Mateo et al. 2015). Genes associated with these peaks were enriched in several notable gene ontology (GO) categories, including Notch and TGF-β signaling, stem cell maintenance, and methyltransferase/histone methyltransferase function (Supplemental Fig. S4). Mitochondrial GO terms were also identified, consistent with reports of a role for FoxG1 in the regulation of mitochondrial function (Pancrazi et al. 2015).

We next examined the intersection of newly defined FOXG1 peaks with the 16,683 sites previously reported as bound by Sox2 in cultured mouse neural progenitors (Lodato et al. 2013). There was a substantial overlap, with 3856 of the 6897 FOXG1 peaks also represented in the Sox2 data set (Fig. 4B). The associated set of genes is strongly enriched for GO categories, including Notch signaling, the histone methyltransferase complex, the mitotic cell cycle checkpoint, and stem cell maintenance (Fig. 4C). This is consistent with the functional consequences of overexpression of FOXG1/SOX2 (namely, cell cycle re-entry and dedifferentiation) and suggests that both factors may play a role in controlling these processes.

**Figure 5.**
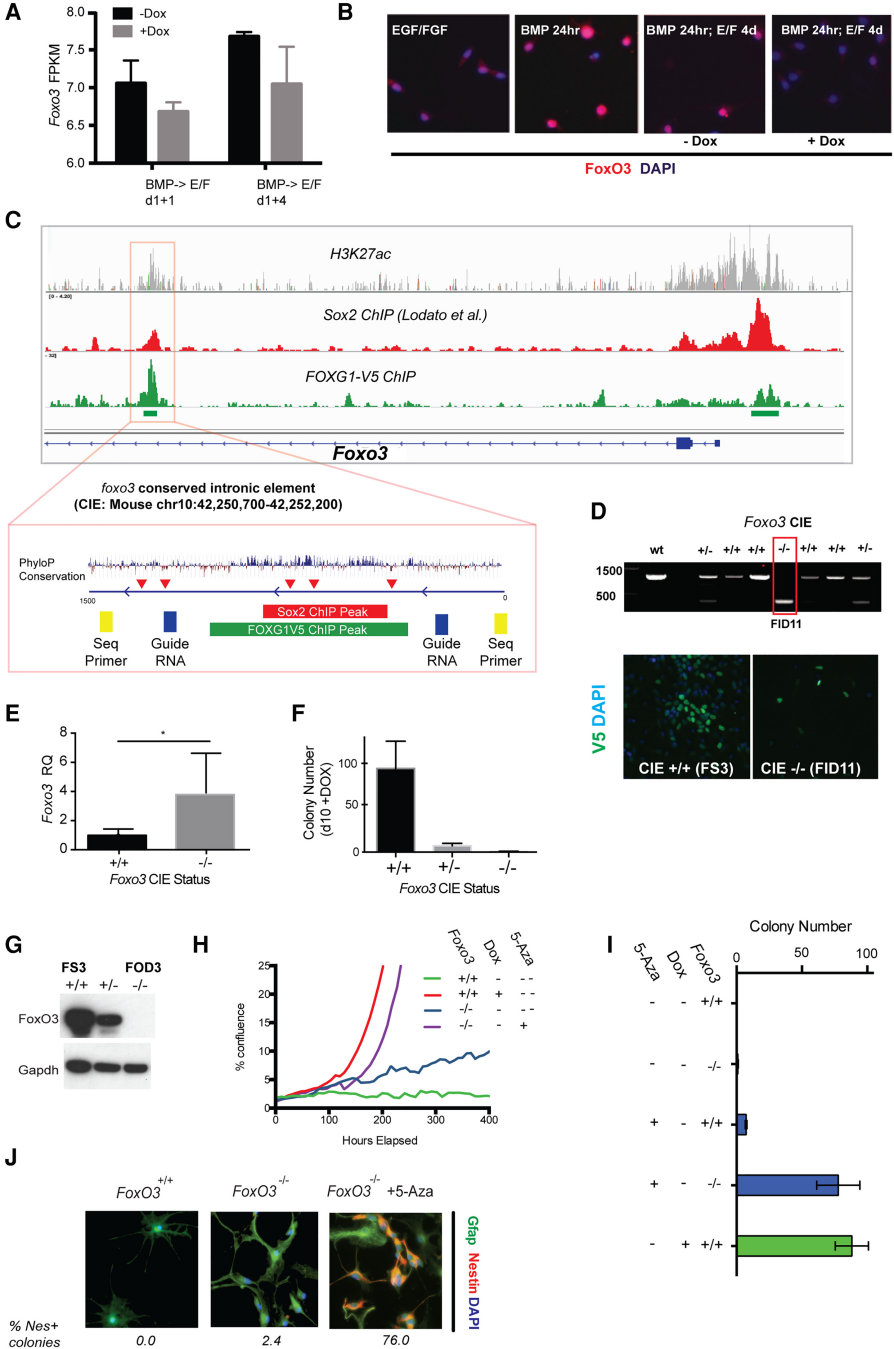
FOXG1/SOX2 forced expression drives reduced expression of Foxo3, and genetic ablation of *Foxo3* removes a barrier to cell cycle re-entry. (*A*) RNA-seq data for Foxo3 following return to EGF/FGF-2 for 1 or 4 d with or without Dox. (FPKM) Fragments per kilobase of transcript per million mapped reads. (*B*) ICC for FoxO3 protein in FS3 cells plated at clonal density after 24 h in EGF/FGF-2, 24 h in BMP4, and return to EGF/FGF-2 for 4 d with or without Dox. (*C*) The *Foxo3* locus is bound by FOXG1 and Sox2 at both the promoter region and a CIE (indicated by red box). (*Top*) These regions enrich for H3K27 acetylation, a marker of active promoters and enhancers, and demonstrates high conservation across mammalian species (PhyloP). Clusters of the AAACA sequence comprising part of both Forkhead- and Sox-binding motifs are indicated by red arrowheads. Guide RNAs flanking the CIE were selected with a view to excision of this region by CRISPR/Cas9 (blue rectangles), along with sequencing primers for genotyping the resulting clones (yellow rectangles). (*D*) PCR genotyping to confirm biallelic deletion with the expected single band in one line (termed FID11); FID11 retains the ability to respond to Dox and hence induce FOXG1-V5 expression, as determined by ICC (*below*). (*E*) Deletion of the FOXG1/SOX2-bound CIE results in derepression of *Foxo3* mRNA expression in NS cell proliferation conditions. *n* = 3. (*) *P* < 0.02). (*F*) Colony formation following Dox-induced FOXG1/SOX2 expression is abolished in CIE-deleted cells. Mean ± SEM. (*G*) Western blot confirming the absence of FoxO3 protein expression in FOD3, a clonal cell line harboring a frameshift insertion–deletion (indel) mutation on the nontargeted allele. (*H*) Following BMP treatment, *Foxo3*^−/−^ FOD3 cells divide slowly in growth conditions (doubling time ∼6 d), in contrast to *Foxo3*^+/+^ controls, which remain cycle-arrested. FOXG1/SOX2 induction or treatment of FOD3 cells with 5-azacytidine (5-Aza) drives rapid colony formation and proliferation to confluence (doubling time ∼24 h). (*I*) Colony-forming assay at 10 d for dedifferentiation responses in *Foxo3*^−/−^ cells and those treated with 5-Aza with and without Dox. (*J*) ICC for Nestin and Gfap. The proportion of cells positive for nestin in representative colonies is indicated *below* the panels. See also Supplemental Figure S5D.

On its own, binding of a TF does not provide evidence of a functional role in regulating the candidate target gene. RNA-seq was therefore performed in order to identify a subset of candidate FOXG1/SOX2-regulated loci (Fig. 4D). As anticipated, BMP exposure rapidly led to down-regulation of *Nestin* expression and up-regulation of the astrocyte markers *Aqp4* and *Gfap*. Of note, FOXG1/SOX2-bound targets that showed altered expression 4 d after Dox treatment and return to self-renewal medium (EGF/FGF-2) included core regulators of the cell cycle (*Plk1*, *Foxo3*, and *Mycn*) and epigenetic processes (*Dnmt1* and *Tet3*) (Fig. 4D). *Foxo3* expression was one of the most significantly up-regulated genes after 24 h of BMP treatment and was down-regulated upon treatment with Dox and exposure to EGF/FGF-2. Foxo3 is a well-established negative regulator of cell proliferation downstream from the PI3K signaling pathway. FOXG1/SOX2-bound regions included the proximal promoter and a conserved intronic element (CIE) harboring multiple motifs for SOX and FOX (Fig. 5C). We therefore pursued this as a candidate functionally important target.

### Transcriptional repression of *Foxo3* by FOXG1/SOX2 removes a barrier to astrocyte cell cycle re-entry

*Foxo3* has an established role in NS cell homeostasis and quiescence (Webb et al. 2013), and a recent study suggests that it is directly regulated by Foxg1 (Vezzali et al. 2016). Our own RNA-seq data indicated a rapid up-regulation of *Foxo3* mRNA following BMP-induced astrocyte differentiation (Fig. 4D). Levels of *Foxo3* mRNA are reduced following addition of Dox and a switch to NS cell medium (Fig. 5A). Immunocytochemistry (ICC) for Foxo3 protein confirmed up-regulation and nuclear translocation following BMP treatment (Fig. 5B). ChIP-seq data indicated binding of both FOXG1 and SOX2 at a highly conserved intronic element within *Foxo3* (Fig. 5C). This region contains multiple repeats of the sequence AAACA, which comprises part of binding motifs for FOX and SOX TFs in NS cells (Lodato et al. 2013).

To directly test the functional significance of binding at the *Foxo3* CIE, we took advantage of CRISPR/Cas9 genome editing, which we optimized for mouse and human NS cells (Bressan et al. 2017). Using a pair of guide RNAs (gRNAs), we deleted the 780-base-pair (bp) Foxg1/Sox2-bound CIE in FOXG1/SOX2-overexpressing FS3 cells (Fig. 5C). Subclones were identified in which both alleles were disrupted (Fig. 5D). Deletion of this element led to increased levels of *Foxo3* mRNA expression under self-renewal conditions (EGF/FGF-2) (Fig. 5E), and proliferation of this line was marginally slower (data not shown). Importantly, these cells were now unable to undergo dedifferentiation in response to FOXG1/SOX2 overexpression (Fig. 5F). We surmise that this regulatory element is critical in enabling FOXG1/SOX2 to repress *Foxo3* expression, thereby removing a critical blockade to cell cycle re-entry.

To confirm the potential relevance of these findings to human GBM, we performed ChIP-seq for FOXG1 in four independent human GNS cell lines (G7, G14, G25, and G166) using a newly generated antibody against endogenous FOXG1. Although less specific than V5 ChIP, we identified a total of 7499 peaks and noted strong enrichment for the forkhead box and related motifs (Supplemental Fig. S5A). These data showed that FOXG1 was bound to the *FOXO3* CIE (Supplemental Fig. S5B).

### Reacquisition of the proliferative NS cell state can be achieved by combined loss of *Foxo3* and alterations to DNA methylation

To test the consequences of *Foxo3* deletion, we excised exon 2 of *Foxo3* in FS3 cells using CRISPR/Cas9-assisted gene targeting (Bressan et al. 2017). Biallelic mutant lines were generated through simultaneous replacement of one *Foxo3* allele with an EF1a-puromycin resistance cassette and insertion–deletion (indel) mutations on the remaining allele (Supplemental Fig. S5C). Foxo3 protein was undetectable in a clonal line that contained a frameshift indel mutation and generated a nonsense product (FOD3) (Fig. 5G). These FOD3 *Foxo3*^−/−^ mutant cells retained a responsiveness to BMP treatment similar to that of their parental cells, with concomitant up-regulation of astrocyte markers (including Gfap) and acquisition of the characteristic morphology (data not shown). However, in contrast to parental controls, which exited cell cycle, *Foxo3* mutant cells proliferated slowly on re-exposure to EGF/FGF-2 without Dox (doubling time of ∼6 d) (Fig. 5H). Thus, *Foxo3* ablation sensitizes astrocytes to growth factors and relieves a barrier to cell cycle re-entry. Importantly, however, these cells did not fully dedifferentiate and retained Gfap expression (Fig. 5H–J). They remained slow-cycling. We conclude that cell cycle entry and differentiation status are uncoupled in the context of *Foxo3* deletion. Additional target genes are therefore required to trigger dedifferentiation and rapid proliferation.

We reported previously that human GBM stem cells fail to undergo terminal differentiation commitment and have aberrant DNA methylation patterns in response to BMP treatment (Carén et al. 2015). Shared transcriptional targets of FOXG1/SOX2 included several regulators of DNA and histone methylation. These genes represent clear candidates that might be involved in destabilizing astrocyte differentiation. Inhibition of DNA methyltransferase activity by the nucleoside analog 5-azacytidine (5-Aza) has been reported to facilitate induced pluripotent stem cell reprogramming (Mikkelsen et al. 2008). We therefore hypothesized that Dnmt inhibition by 5-Aza might facilitate dedifferentiation by interfering with the establishment or maintenance of the DNA methylation profile in differentiating astrocytes. Indeed, either 5-Aza or ascorbic acid (a cofactor for Tet proteins) could trigger increased proliferation in populations of *Foxo3* mutant astrocytes (Supplemental Fig. S5D). This was quantified for 5-Aza using colony formation assays for the slow-cycling BMP-treated Foxo3 mutants (FOD3). Strikingly, the combination of 5-Aza treatment with *Foxo3* deletion resulted in the emergence of rapid-cycling populations forming numbers of Nestin-positive colonies similar to the Dox-treated FS3 cultures (Fig. 5H–J). Thus, 5-Aza in combination with loss of *Foxo3* can phenocopy the effects of FOXG1/SOX2 induction. Resetting of DNA methylation patterns that are acquired during astrocyte differentiation may therefore be a critical feature of FOXG1/SOX2 reprogramming activity.

### FOXG1 overexpression affects multiple regulators of DNA methylation to facilitate dedifferentiation

**Figure 6.**
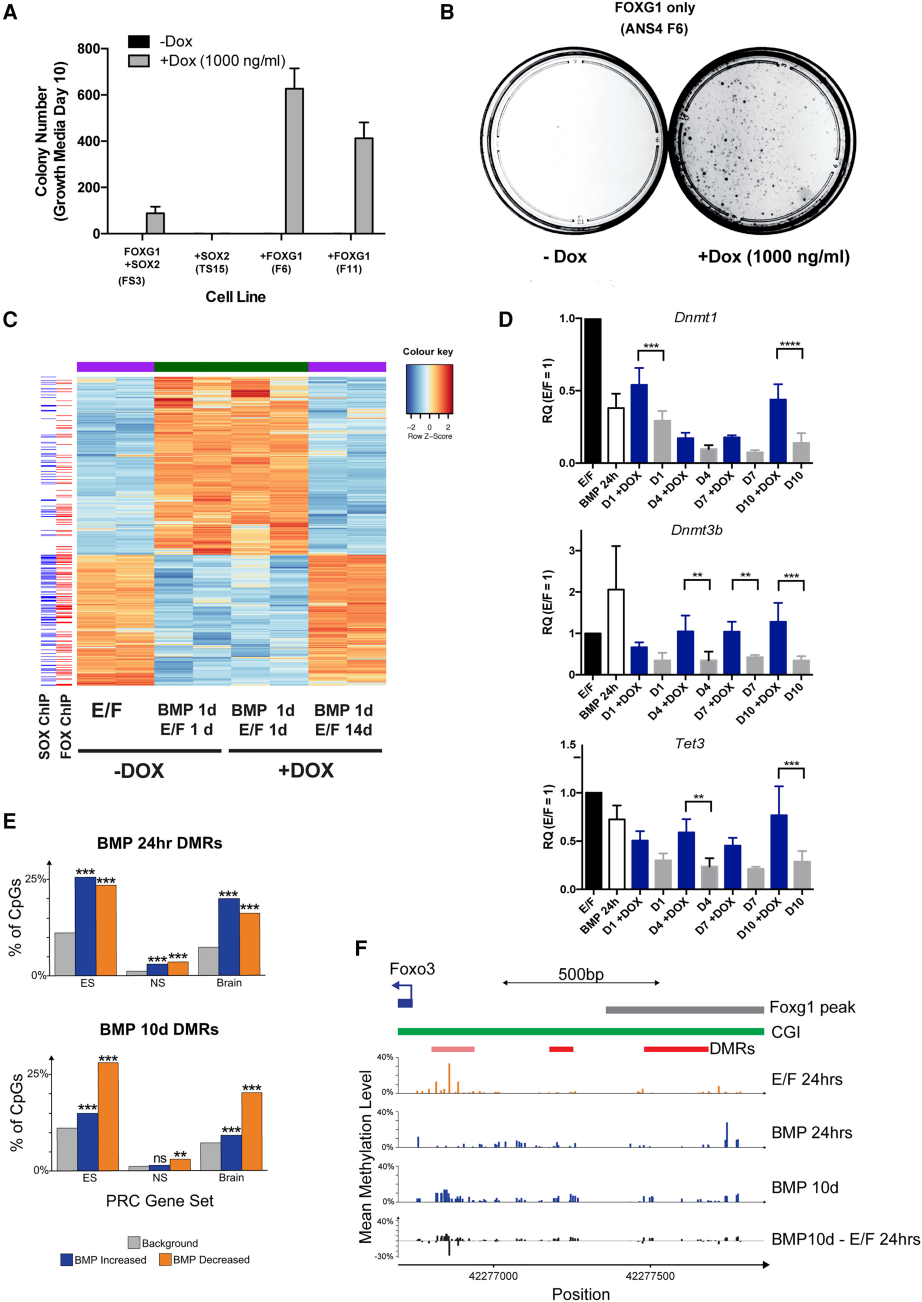
FOXG1 overexpression results in increased activation of regulators of DNA methylation, and these may affect key polycomb target genes. (*A*) Colony numbers upon return to self-renewal medium with or without 1000 ng/mL Dox for 10 d following 24 h of BMP4 treatment. Induction of FOXG1 alone in two independent lines (F6 and F11) induced colony formation at higher efficiency than in FS3. Induction of SOX2 alone (TS15) was not sufficient to drive colony formation. (*B*) Example of a colony-forming assay for F6 showing colonies after 10 d in EGF/FGF-2 only on the addition of Dox. (*C*) RNA-seq confirms that, following FOXG1 induction by Dox, BMP4-treated F6 cells reacquire an NS cell-like transcriptional signature. (*Left*) Alignment with ChIP-seq data for FOXG1 and SOX2 indicates that many of the genes activated on dedifferentiation are bound by FOXG1 and SOX2. (*D*) qRT–PCR analysis of *Dnmt1*, *Dnmt3b*, and *Tet3*. Mean ± SD. *n* = 4. Significance was assessed by two-way ANOVA with Bonferroni post-hoc test. (**) *P* ≤ 0.01; (***) *P* ≤ 0.001; (****) *P* ≤ 0.0001. (*E*) Analysis of enrichment of reduced representation bilsulfite sequencing (RRBS) identified differentially methylated regions (DMRs) near genes marked by polycomb in mouse embryonic stem (ES) cells, NS cells, and brains. Shown is the percentage of CpGs assayed by RRBS found near polycomb-marked genes (background, gray) compared with those in significant DMRs after either 24 h or 10 d of differentiation. (Blue) BMP-increased methylation; (orange) BMP-decreased methylation. Significance was assessed with Fisher's exact tests (**) *P* < 0.01; (***) *P* < 0.001. *n* = 3. (*F*) Mean methylation profiles observed by RRBS in the *Foxo3* promoter, including the locations of its CpG island (CGI) and Foxg1 ChIP-seq peak. Significant DMRs are shown in red together with an additional DMR that did not reach statistical significance in all replicates of the experiment (pale red).

We next investigated the effect of forced expression of higher levels of FOXG1 or SOX2 alone using the F6 and S15 lines, respectively (Supplemental Fig. S2E,F). Each of these lines enabled higher levels of each individual factor to be expressed in differentiating astrocytes. High levels of induction of FOXG1 alone, but not SOX2, were sufficient to drive efficient colony formation in two independent FOXG1-inducible lines (F6 and F11) (Fig. 6A,B). The resulting dedifferentiated cells displayed morphology, proliferation kinetics, and marker expression similar to the parental line and responded to BMP-induced differentiation (Supplemental Fig. S6A,C–E). RNA-seq confirmed that these cultures were reacquiring NS cell-like transcriptional signatures, and many of the activated genes included FOXG1/SOX2-bound genes (Fig. 6C). We confirmed by RNA-seq and quantitative RT–PCR (qRT–PCR) that there is a significant increase in expression of *Dnmt1*, *Dnmt3b*, and *Tet3* following increased FOXG1 expression (Fig. 6D; Supplemental Fig. S6B).

### DNA methylation changes at polycomb target genes, including *Foxo3*, occur during astrocyte differentiation

To define the DNA methylation changes that accompany BMP-induced astrocyte differentiation, we performed reduced representation bilsulfite sequencing (RRBS). Analysis of the resulting methylation profiles identified a total of 3231 significantly differentially methylated regions (DMRs) after 24 h or 10 d of BMP-induced differentiation (756 with reduced methylation and 2475 with increased methylation). These DMRs were significantly enriched near developmental TFs (Supplemental Fig. S6F). Developmental TFs are known to be regulated by polycomb-repressive complexes; indeed, BMP-induced DMRs were enriched near polycomb-repressive complex target genes previously reported in mouse NS cells, embryonic stem cells, and brains (Fig. 6E; Meissner et al. 2008). This included methylation changes at the promoter of *Foxo3* close to a Foxg1-binding site (Fig. 6F). These analyses suggest that DNA methylation changes occur at developmental TFs during astrocyte differentiation and that FOXG1 may help in reconfiguring these during dedifferentiation via its control of multiple regulators of DNA methylation.

### Genetic ablation of *FOXG1* in human GNS cells does not affect in vitro proliferation, but *SOX2* is essential

**Figure 7.**
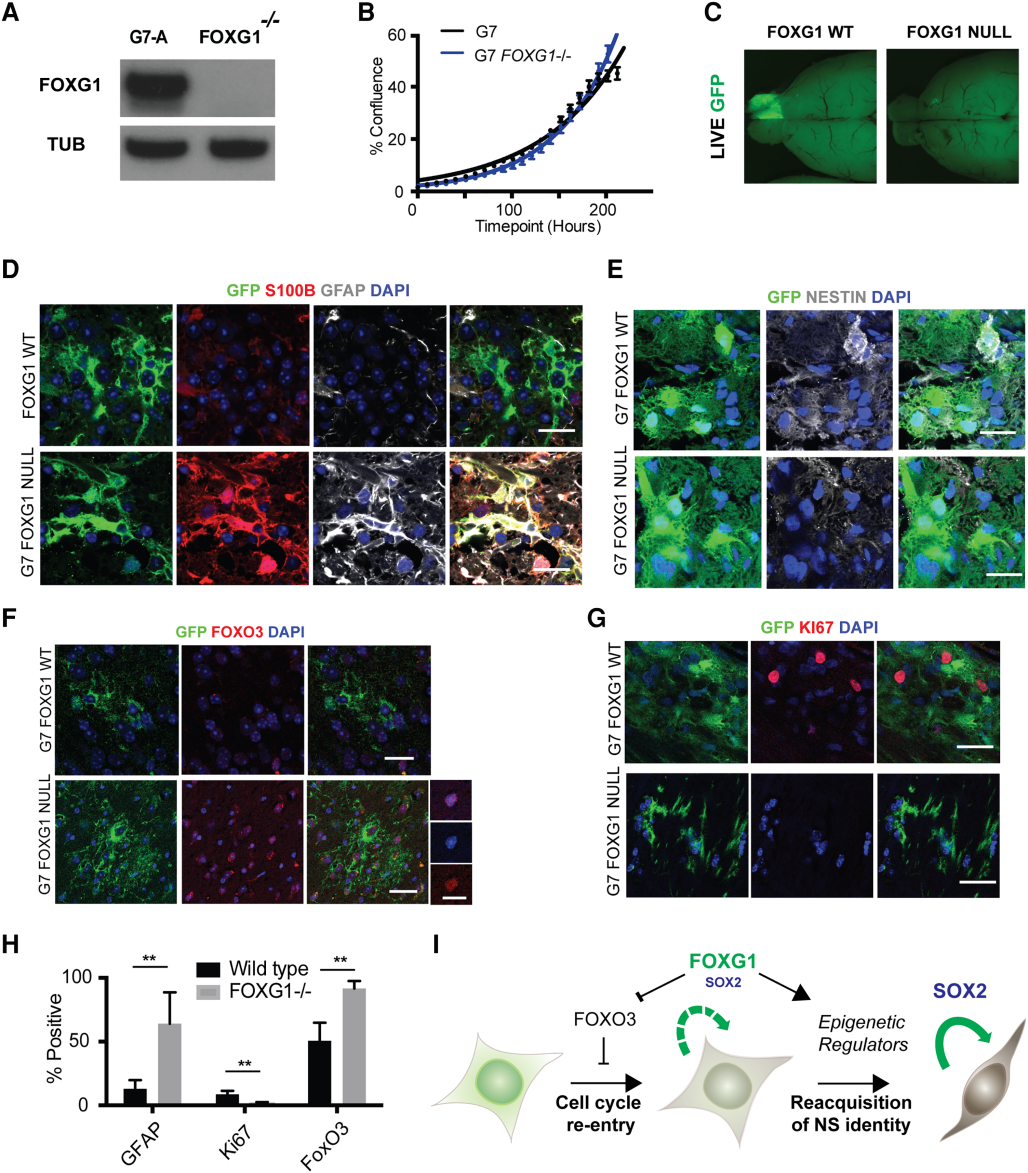
Genetic ablation of FOXG1 in human GBM stem cells using Cas9-assisted gene targeting. (*A*) CRISPR/Cas-based gene targeting was used to knock out FOXG1 in G7 cells, and no protein was detectable by Western blot, with a frameshift mutation demonstrated on the second FOXG1 allele in this clone (see Supplemental Fig. S7). (*B*) Growth curve displaying percentage confluence over time for G7 and G7 *FOXG1*^−/−^ cell lines, indicating that the *FOXG1*^−/−^ clone proliferates at a rate similar to that of parental controls in vitro*.* (*C*) Upon xenotransplantation, wild-type G7 cells expressing a GFP reporter form invasive tumors, but *FOXG1*^−/−^ derivatives fail to do so. *n* = 4 for each cell line. (*D*) Immunohistochemistry (IHC) analysis of xenografts reveals that the G7 FOXG1 mutant cells display increased expression of astrocyte markers S100β (red) and GFAP (gray), reduced expression of NESTIN (gray) (*E*), increased expression of FOXO3 (*F*), and decreased expression of Ki67 (red) (*G*). (*H*) Quantitation of the percentage of cells positive for GFAP, Ki67, and FOXO3 from IHC. (*I*) Working model of FOXG1 and SOX2 function in GBM based on this study. (Green cell) Post-mitotic or quiescent astrocytes; (brown/gray cell) radial glia-like proliferative NS cell. Bar, 10 µm; bar for higher-magnification images in *F*, 20 µm. Students *t*-test, *n* = 4; *P* < 0.005.

Previous studies using shRNA knockdown of FOXG1 have suggested an important role in promoting tumor growth (Verginelli et al. 2013). CRISPR/Cas9 provides new opportunities for decisive functional genetic studies in primary human GBM stem cells. Using recently optimized protocols (Bressan et al. 2017), we next performed gene targeting via homologous recombination to delete *FOXG1* in human primary GNS cells (G7) (Supplemental Fig. S7A). One of the resulting clonal lines (G7-A) harbored a 23-bp frameshift insertion at the second allele and demonstrated loss of FOXG1 protein by immunoblotting (Fig. 7A; Supplemental Fig. S7A). In contrast to previously reported findings using tumor sphere models, we found no discernible effect of FOXG1 ablation on proliferation rates of GNS cells in vitro (Fig. 7B).

We next compared the FOXG1 loss-of-function phenotype with SOX2 loss in G7 cells. Previous studies have suggested that Sox2 is required for self-renewal of forebrain NS cells: Homozygous knockout by conditional deletion or CRISPR/Cas9 targeting is incompatible with colony formation (Gómez-López et al. 2011; Bressan et al. 2017). Here, CRISPR/Cas9 was used to mutate the single coding exon of *SOX2* (Supplemental Fig. S7B). We were unable to recover expandable SOX2 mutant clones, suggesting that these may have a proliferation defect. The proportion of SOX2-negative cells was tracked in the primary transfected population over time by ICC (Supplemental Fig. S7C–E). Approximately 25% of mutant cells were detectable in the transfected population at day 7; however, by day 14 and day 42, this subpopulation had dropped to ∼18% and <1% of the population, respectively. Coculture with the nondeleted wild-type cells clearly could not rescue the proliferation defect. We conclude that loss of *SOX2* ablates the proliferative capacity of patient-derived GBM cells in a cell-autonomous manner. This is in contrast to *FOXG*1, which is dispensable for in vitro NS cell proliferation.

### *FOXG1* mutant human GNS cells are sensitized to cytostatic signals in vivo and up-regulate FOXO3

To test the consequences of FOXG1 loss in vivo, we transplanted cells orthotopically into the brains of immunocompromised mice. A GFP reporter construct was inserted at the safe harbor *AAVS1* locus in both parental control cells and the *FOXG1*^−/−^ clone to enable monitoring of cells following xenotransplantation. Consistent with the previously reported shRNA knockdown results (Verginelli et al. 2013), we saw a failure of the *FOXG1*^−/−^ G7-A cells to form tumors on transplantation into immunocompromised mice (*n* = 4) (Fig. 7C). We hypothesized that FOXG1 is able to protect cells from prodifferentiation signals that would trigger exit from the cell cycle in vivo.

Our findings in mouse NS cells suggested that FOXG1 operates in part by helping repress *FOXO3*, and this could be a key effector of its function by limiting astrocyte differentiation. We therefore assessed expression of GFAP and FOXO3 in the FOXG1 knockout cells following transplantation in vivo. The transplanted cells were present at the injection site, and these were found to express high levels of GFAP and FOXO3 and low levels of Ki67 compared with wild-type controls. They also displayed morphological features of differentiated “star-shaped” astrocytes (*n* = 4) (Fig. 7D–H). This indicates that FOXG1 is required to sustain GNS cell growth in vivo. In conclusion, we found that SOX2 is essential for continued proliferation of GBM stem cells, while FOXG1 is not. However, increased levels of FOXG1 safeguard the stem cell state from prodifferentiation cues encountered outside the endogenous SVZ niche. This restriction of differentiation commitment is mediated at least in part through repression of negative regulators of proliferation such as *FOXO3.* (Fig. 7I).

## Discussion

There are important conceptual and mechanistic similarities between cellular transformation in human cancers and cellular reprogramming (Suvà et al. 2013). FOXG1 and SOX2 are key regulators of forebrain neural progenitor fate and are known reprogramming factors (Lujan et al. 2012). Here we demonstrated that high FOXG1 and SOX2 levels, a consistent feature of GNS cells, are functionally important in driving a highly proliferative, growth factor-responsive, radial glia-like NS cell state. These master regulators operate through transcriptional control of various stem cell-associated pathways, most notably cell cycle and epigenetic regulators. Cancer stem cells therefore deploy overexpression of key lineage-affiliated TFs as a mechanism to fuel their self-renewal—the same strategy used by stem cell biologists in experimental reprogramming.

FOXG1 is consistently up-regulated across all GNS cells that were assessed. Using ATAC-seq profiling of human GNS and NS cells, we identified an enrichment of open regions containing many neurodevelopmental TF motifs, including binding sites of SOX and FOX TF families. This supported our hypothesis that increased levels of FOXG1 and SOX2 might be important in driving GBM cell self-renewal and is consistent with the known roles of these factors during development of the mammalian forebrain (Xuan et al. 1995).

We initially explored Foxg1 loss of function using a new conditional NS cell line. Mutant cells become sensitized to differentiation cues, but, surprisingly, there was no proliferative defect in vitro. This is in contrast to loss of Sox2, which has been shown previously to be a critical factor for proliferation of mouse NS cells. This suggested to us that the gain-of-function phenotype for Foxg1 is more critical, and its role might be specifically in limiting terminal differentiation commitment or driving dedifferentiation. A quantitative colony formation assay was developed to explore the consequences of increased expression of human FOXG1 and SOX2 in dedifferentiating astrocytes, thereby mimicking the increased levels of FOXG1 seen in GBMs. We did not observe increased levels of SOX2 protein in GNS cells compared with NS cells. However, unlike FOXG1, SOX2 is amplified in GBM. It is possible that the levels of SOX2 in NS cells are already saturating in vitro. We found that NS cells plated at low density and treated with BMP4 for 24 h exit cell cycle with acquisition of astrocyte morphology and markers. Quiescent NS cells in vivo express Gfap. So, are we modeling the transition from quiescence to reactivation/proliferation or terminal differentiation to dedifferentiation? We could induce NS cell colony formation by FOXG1/SOX2 induction when using fresh primary postnatal astrocyte preparations. Furthermore, we found recently that low-density BMP-treated astrocytes have reduced levels of quiescent stem cell astrocyte markers (data not shown). A key functional criterion for distinguishing quiescent astrocytes and differentiating astrocytes is that the latter cannot be driven into cycle when re-exposed to EGF/FGF-2. Thus, we view our assay as a dedifferentiation response.

Our ChIP-seq data for FOXG1 and the intersection with SOX2-bound sites suggested that these factors have common target genes, including both important core cell cycle and epigenetic regulators. However, we found no indication of physical interaction between SOX2 and FOXG1 using protein coimmunoprecipitation (data not shown). This is consistent with characterized SOX2 protein partner analysis in mouse NS cells (Engelen et al. 2011). Rather, it seems likely that FOXG1 and SOX2 are cooperating indirectly at the gene regulatory network level.

Exposure to Dox and EGF/FGF-2 triggered a relatively rapid emergence of proliferating colonies, whether from NS cell-derived in vitro generated astrocytes or astrocytes from primary cultures. Inspection of this response by time-lapse imaging together with the sizes of resulting colonies suggested that cell cycle re-entry was an early event. We recognized that multiple targets will contribute to the potency of FOXG1/SOX2 activity and searched for those candidates that might have a major contribution. Foxo3, which has an established role in NS cell homeostasis and quiescence, emerged as a functionally important transcriptional target of FOXG1/SOX2. This finding is consistent with Foxo3 as a transcriptional target of Foxg1 during telencephalic development (Vezzali et al. 2016). FOXO3 activity is also known to be affected by interaction with FOXG1 at the protein level (Seoane et al. 2004); FOXG1 therefore exerts a dual inhibition of FOXO3 activity: at the protein–protein level and through transcriptional suppression. We used CRISPR/Cas9 genetic ablation to confirm FOXG1 repression of *Foxo3* at the transcriptional level. Importantly, in the absence of the FoxG1-bound repressive element in the *Foxo3* intron, NS cells could no longer respond to Dox. Therefore, transcriptional repression of Foxo3 through this site may be the primary mechanism of control by FoxG1, with the sequestration through protein–protein interaction being an added layer of regulatory control.

*Foxo3* ablation removes a barrier to cell cycle re-entry; however, the mutant cells retained astrocyte morphology and high GFAP expression and displayed slow proliferation kinetics on the restoration of growth factors following BMP treatment. Foxo3 repression alone is therefore insufficient to trigger full dedifferentiation to an NS cell-like state. Additional targets must exist. Given the prominence of methyltransferase and histone methyltransferase complexes in GO analysis of the FOXG1/SOX2-bound regions, we explored whether resetting of DNA methylation patterns could remove a barrier to dedifferentiation. This proved to be the case, as a short 24-h pulse of a low dose of 5-Aza (a nucleoside analog that inhibits DNA methyltransferase activity) or ascorbic acid (a cofactor of the TET family of enzymes that trigger DNA demethylation) was sufficient to stimulate rapid proliferation of *Foxo3* mutant cells. Thus, the effects of FOXG1/SOX2 overexpression can be phenocopied by removal of Foxo3 and reconfiguration of DNA methylation patterns. Multiple regulators of DNA methylation were bound by FOXG1, including Tet3, Dnmt3b, and Dnmt1. These displayed changes in expression upon Dox treatment in FOXG1-alone-overexpressing cells (F6).

DNA methylation profiling using RRBS identified significant methylation changes in astrocytes following 24 h of BMP4 treatment that were heavily enriched for polycomb target genes, including Foxo3. Unfortunately, as only a subpopulation of the cells undergoes dedifferentiation following re-exposure to growth factors and addition of Dox, we were unable to identify any significant changes in methylation after 4 d (data not shown). Future studies will require isolation/enrichment for the earliest dedifferentiating cells to define the specific link between key sites of methylation changes and FOXG1 binding. Tet3 is a clear candidate that might impact the stability of the methylome in differentiating astrocytes. Our current data support a model in which high levels of FOXG1/SOX2 have at least two complementary activities: stimulation of core cell cycle regulators and triggering of epigenetic resetting to drive post-mitotic astrocytes into the more immature radial glial-like NS cell state (Fig. 7I). Further definition of the downstream targets of these factors might uncover “druggable” targets and guide rational combination therapy strategies.

Not all astrocytes are able to respond to FOXG1/SOX2. It is possible that additional factors or signaling pathway manipulations could improve efficiency. There might also be some stochastic element to triggering dedifferentiation, as is the case with induced pluripotent stem cell reprogramming (Buganim et al. 2012). Other noteworthy annotated gene sets that we identified via ChIP-seq analysis included mitochondrial function, Notch, and Wnt/β-catenin signaling. Many of these have been implicated in the growth of GBMs, and further studies will be needed to define whether these can enhance dedifferentiation.

Using CRISPR/Cas9 gene targeting, we were able to genetically ablate FOXG1 in primary human GBM stem cells. FOXG1 is dispensable for in vitro NS cell proliferation when cultured in adherent conditions with EGF/FGF-2. This seemingly contradicts previous shRNA knockdown studies that concluded that FOXG1 is required to sustain proliferation (Verginelli et al. 2013). However, Verginelli et al. (2013) assayed proliferation using tumor spheres, a condition in which spontaneous differentiation can occur. Thus, the discrepancy is likely explained by differences in culture regimes. These findings are also consistent with the fact that we can routinely derive NS cell lines from different regions of the developing nervous system (midbrain, hindbrain, and spinal cord) that do not express *FoxG1* either in vivo or in vitro. Thus, FOXG1 is not an essential cell cycle driver in NS cells; rather, it is required to protect cells from prodifferentiation cues and can trigger the transition out of the nonproliferative state.

Previous studies have explored the core transcriptional circuits that might be exploited by GBM stem cells. A reprogramming cocktail incorporating SOX2, OLIG2, and POU3F2 has been used to reinstate tumorigenicity in “differentiated” glioblastoma cells (Suvà et al. 2014), and this network was generated by focusing on TFs differentially expressed between GBM stem cells and serum-induced differentiating progeny. FOXG1 was not among the factors comprising the core transcriptional circuit identified in these studies. However, a recent study by the Barres laboratory (Zhang et al. 2016) has identified genes differentially expressed between immature fetal astrocytes and post-mitotic adult cortical astrocytes. FOXG1 is indeed one of the most differentially expressed genes (Supplemental Fig. S7F). We speculate that up-regulation of FOXG1 expression is a critical event in the emergence of GBM, occurring either early in tumorigenesis to produce primary glioblastoma or later, resulting in secondary transformation of a low-grade glioma. In keeping with this, we found variable FOXG1 expression in a panel of tumor lines derived from World Health Organization grade II and grade III gliomas (data not shown).

In conclusion, we show that elevated FOXG1 plays a functionally important role in limiting differentiation commitment. SOX2 is required to sustain NS and GNS cell proliferation. When coexpressed, these two activities drive self renewal and enforce a proliferative radial glial-like NS cell state. Although we found no evidence of a protein level interaction between these factors, they share multiple core cell cycle and epigenetic regulatory targets. Our findings highlight the increasing evidence in support of a critical role for neurodevelopmental TFs in driving unconstrained self-renewal in GBM.

## Materials and methods

### Cell culture

Mouse and human NS and GNS cell lines were derived from adult SVZ, fetal cortex, or primary glioblastoma specimens as described previously (Conti et al. 2005; Sun et al. 2008). Established lines were cultured in serum-free basal medium supplemented with N2 and B27 (Life Technologies), 1 mg/mL laminin (Sigma), and 10 ng/mL growth factors EGF and FGF-2 (Peprotech). Medium was changed every 3 d, and cells were split typically once per week after dissociation with Accutase solution (Sigma) and centrifugation.

BMP treatment comprised plating dissociated NS cells at low density (10 cells per square millimeter) in medium supplemented with 10 ng/mL BMP4 (Peprotech) in place of EGF/FGF-2. After 24 h, this was replaced by standard growth medium containing EGF/FGF-2. Colonies were stained with ethidium bromide and visualized under UV light.

IENS cells, described previously (Bruggeman et al. 2007), were kindly provided by M. Van Lohuizen (Nederlandse Kappersakademie, Amsterdam). Supplemental Table 1 details the mouse NS cell line derivatives established here and summarizes their differentiation/dedifferentiation characteristics. Growth curves were generated using an IncuCyte live-cell imaging system.

Primary mouse astrocyte cultures were prepared from the trypsin-digested cortical plate tissue of postnatal day 3 (P3) mouse cortices (strain MF1), according to established protocols (Schildge et al. 2013), including shake-off after 1 wk to remove contaminating microglia and progenitor cells.

### Derivation of stable transgenic and knockout cell lines

One million cells were transfected with the Amaxa Nucleofector system using either the X005 pulse protocol (human cells) or T-030 protocol (mouse cells).

For inducible transgene overexpression, a total of 6 µg of DNA was supplied, comprising piggyBac transposase pBASE, pCAG-rtTA(Tet3G), and pDEST-TetOn vector in 1:1:2 ratios. For CRISPR targeting, gRNAs (times two), targeting vector (where appropriate), and Cas9 nickase were transfected in a 1:1:1:2 ratio.

Cells were plated in 10-cm dishes, with Dox added after 24 h where appropriate, and selection commenced 48 h after transfection using 5 µg/mL blasticidin, 1 µg/mL puromycin, or 100 µg/mL hygromycin. Each of these antibiotics produced uniform cell death within 7 d in untransfected mock controls (both human NS and GNS cells).

G7 primary human GNS cells were transfected with Cas9 nickase, gRNAs corresponding to the forkhead domain of the *FOXG1* locus, and a targeting vector comprising an EF1a-puromycin antibiotic resistance cassette flanked by 1-kb homology arms specific for the locus.

### ICC

Cells were washed with PBS and fixed using 4% paraformaldehyde for 10 min at room temperature. Samples were incubated overnight with primary antibodies in blocking solution (PBST + 3% goat serum and 1% BSA) followed by incubation with appropriate secondary antibodies and 4′,6-diamidino-2-phenylindole (DAPI). Images were obtained using a Zeiss Observer Z1 microscope and AxioVision software or a PerkinElmer Operetta high-content imaging system and Harmony software. Transplanted mouse brains were harvested, sectioned into 30-µm slices using a vibratome, stained using immunohistochemistry (IHC) as free-floating staining, and imaged using a Leica SP8 confocal microscope.

The following primary antibodies were used: Olig2 (1:100; Millipore), V5 tag (1:1000; eBioscience), Sox2 (1:50; R&D Systems), mNestin (1:10; Developmental Studies Hybridoma Bank), hNestin (1:500; R&D Systems), FOXG1 (1:3; hybridoma clone 17B12), FOXO3 (1:800; Cell Signaling Technology), GFAP (1:1000; Sigma), S100 (1:100; DAKO), Stem121 (1:500; Stem Cell Technology), BLBP (1:200; Santa Cruz Biotechnology), and Ki67 (1:500; Lab Vision). EdU incorporation assays were performed as described previously (Carén et al. 2015).

### Western immunoblotting

Immunoblotting was performed using standard protocols. Antibodies were diluted in 5% milk powder in PBS Triton 0.1%, and protein detection was carried out with HRP-coupled secondary antibodies and X-ray films. The following primary antibodies were used: FOXG1 (1:15; hybridoma clone 17B12), SOX2 (1:400; R&D Systems), GAPDH (1:1000; GenTex), and V5 tag (1:1000; eBioscience).

### qRT–PCR and low-density arrays (LDAs)

RNA extraction was performed using the Qiagen RNeasy Plus minispin column kit, eluting in 50 µL of RNase-free water, and using an additional DNase step. RNA concentration was determined using the Qubit RNA High-Sensitivity kit (Life Technologies). Reverse transcription was performed using the Invitrogen SuperScript III kit according to the manufacturer's instructions. TaqMan qPCR and TaqMan LDA card assays were performed using TaqMan Universal PCR Master Mix and assays (Applied Biosystems) according to the manufacturer's guidelines. Results were normalized to the housekeeping gene Gapdh and analyzed with HTqPCR (Dvinge and Bertone 2009). The following TaqMan assays were used: mGapdh (Mm99999915_g1), mFoxG1 (Mm02059886_s1), mFoxo3 (Mm01185722_m1), mGfap (Mm01253033_m1), mAqp4 (Mm00802131_m1), mS100b (Mm00485897_m1), mNestin (Mm00450205_m1), mOlig2 (Mm01210556_m1), mBlbp (Fabp7) (Mm00445225_m1), mSox2 (Mm03053810_s1), mDnmt1 (Mm01151063_m1), mDnmt3b (Mm01240113_m1), mTet3 (Mm00805756_m1), and hGAPDH (Hs02758991_g1).

### RNA-seq library construction

RNA-seq libraries were prepared from 100 ng of mRNA extracted using Qiagen RNeasy kits. Library preparation was conducted using NEBNext mRNA reagents (E6100) and multiplex indices for Illumina (E7335).

### ChIP-seq library construction

Chromatin was prepared and immunoprecipitation was undertaken according to protocols described previously (Sofueva et al. 2013). Sonication was performed in 0.7% SDS using a Diagenode Bioruptor (maximum power 30 sec on and 30 sec off for 45 min). Pull-down was undertaken using Dynabead protein G sepharose beads (Thermo Scientific) conjugated with 10 µL of ChIP-grade antibody (anti-FoxG1 [Abcam, ab18259] and anti-V5 [Abcam, ab15828]) diluted in 250 µL of buffer.

### ATAC-seq library construction

ATAC-seq libraries were prepared using Illumina Nextera reagents as described (Buenrostro et al. 2013), with PCR amplification and indexing using published sequencing adapter primer sequences supplied as oligonucleotides (Sigma) (Buenrostro et al. 2013).

### ChIP-seq data analysis

Filtered read files were imported to the Galaxy Web-based analysis portal. Within Galaxy, the files were parsed into Sanger FastQ format, and then each read was truncated from 100 to 55 bp (base pairs 10–65 of the original read). The read files were each mapped to the mouse genome (mm9 assembly) using Bowtie conFig.d with default parameters. The resulting BAM alignment files were merged into a single file, and peak calling was performed using the MACS 2.0 algorithm. Galaxy was also used to determine motif enrichment (SeqPos Motif tool), and the Stanford University genomic regions of enrichment annotations tool (GREAT version 3.0.0) was used for target gene and ontology analysis.

### ATAC-seq data analysis

ATAC-seq data were normalized and compared as described previously (Carén et al. 2015), with the exception of motif analysis, which was applied to GNS-enriched loci using all accessible chromatin sequences as a control. Heat maps were generated from CQN-normalized data using the Euclidean distance metric and Ward's method for clustering the rows.

### RRBS library preparation

gDNA was isolated from F6 cells using the MasterPure complete DNA purification kit (Epicentre) from three independent experiments and concentrated with the TM-5 DNA Clean and Concentrator kit (Zymo Research) before being quantified by Qubit dsDNA BR assay and Nanodrop. Eighty-five nanograms of each purified DNA sample was processed using the Ovation RRBS Methyl-Seq system kit (NuGEN Technologies). Unmethylated phage λ DNA (0.5 ng) was spiked into each sample to allow assessment of bisulfite conversion efficiency. Briefly, the methylation-insensitive restriction enzyme MspI was used to digest the gDNA, and digested fragments were ligated to adapters. Adapter-ligated fragments were then repaired before bisulfite conversion with the EZ DNA Methylation-Lightning kit (Zymo Research). Bisulfite-treated adapter-ligated fragments were amplified by 15 cycles of PCR and purified using Agencourt RNAClean XP beads. Libraries were quantified using the Qubit dsDNA HS assay and assessed for size and quality using the Agilent Bioanalyzer DNA HS kit. Sequencing was performed using the NextSeq 500/550 high-output version 2 kit (150 cycles; Illumina) on the NextSeq 550 platform. Libraries were combined into equimolar pools and run across four flow cells. Library preparation and sequencing were performed at the Edinburgh Clinical Research Facility.

### Intracranial xenotransplantation

Transplants were performed as described previously (Pollard et al. 2009). Briefly, we used a stereotaxic frame to inject 100,000 cells in 1 µL into the striatum of adult NSG immunocompromised mice (aged 4–8 wk). Coordinates were 1 mm anterior and 2 mm lateral to the Bregma and 2.5 mm deep.

## Supplementary Material

Supplemental Material
