# Supplementary material for: Elevated FOXG1 and SOX2 in glioblastoma enforces neural stem cell identity through transcriptional control of cell cycle and epigenetic regulators
Source: Genes Dev. 2017 Apr 15;31(8):757–73. doi: 10.1101/gad.293027.116 (PMC5435889; doi:10.1101/gad.293027.116)
Supplement: Supplemental Material [file supp_31_8_757__index.html]

Elevated FOXG1 and SOX2 in glioblastoma enforces neural stem cell identity through transcriptional control of cell cycle and epigenetic regulators — Supplemental Material 

# Elevated FOXG1 and SOX2 in glioblastoma enforces neural stem cell identity through transcriptional control of cell cycle and epigenetic regulators

## Supplemental Material

- Supplemental\_Data.pdf
- Supplemental\_table\_2.csv
- Supplemental\_Video.m4v
