# Supplementary material for: Elevated FOXG1 and SOX2 in glioblastoma enforces neural stem cell identity through transcriptional control of cell cycle and epigenetic regulators
Source: Genes Dev. 2017 Apr 15;31(8):757–73. doi: 10.1101/gad.293027.116 (PMC5435889; doi:10.1101/gad.293027.116)
Supplement: Supplemental Material [file supp_gad.293027.116_Supplemental_Data.pdf]

Supplemental information

Supplementary Figures

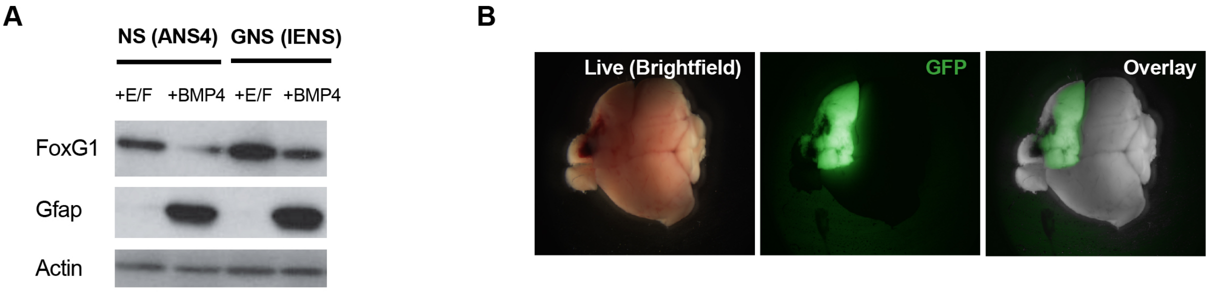

Figure S1 | (A) Immunoblot of Foxg1 in IENS and ANS4 cells. IENS cells express high levels of Foxg1 in self-renewal conditions and after BMP exposure, compared to ANS4 controls. Gfap levels increase on exposure to BMP4 indicating differentiation. Actin was used as a loading control. (B) IENS-GFP labelled cells generate tumours 4–5 weeks after orthotopic xenotransplantation.

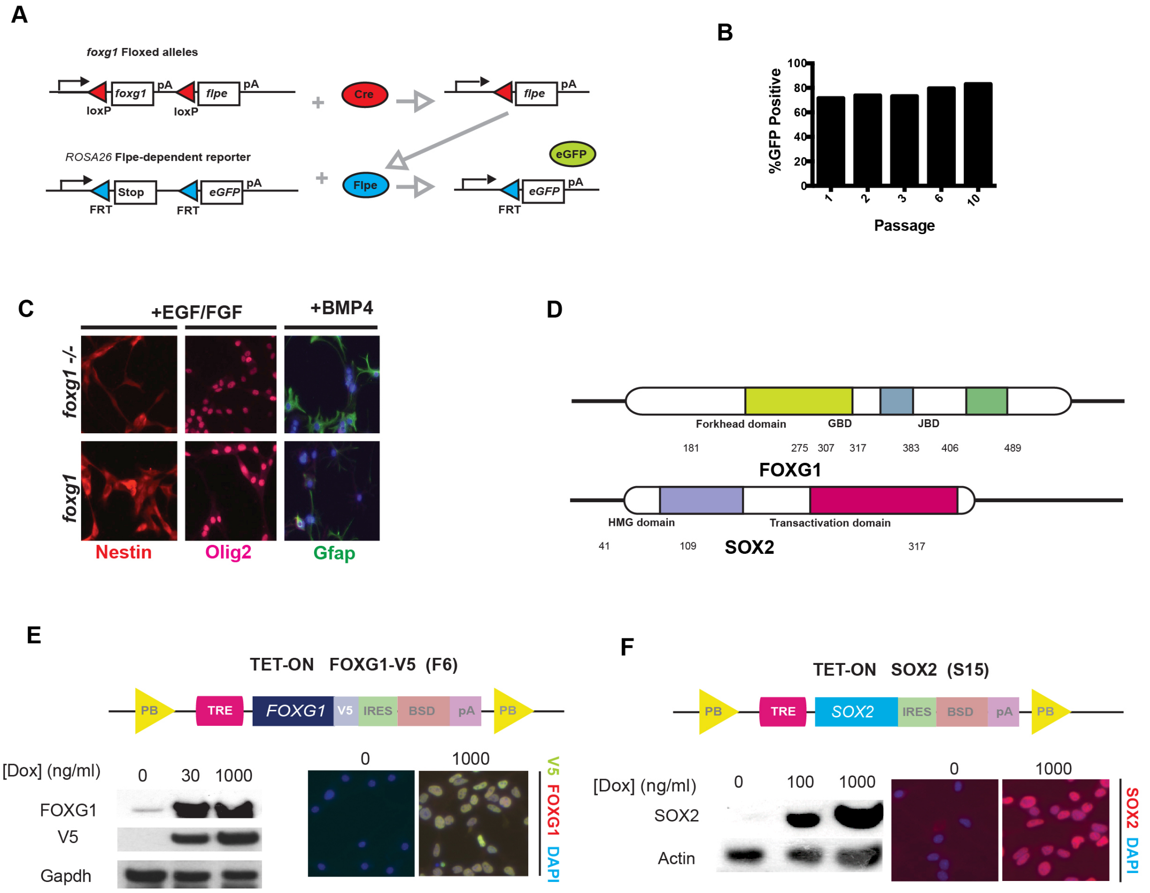

Figure S2 | (A) Strategy for *Foxg1* deletion in *Foxg1*$^{flox/flox}$ NS cells. loxP elements flank the *Foxg1* coding exons and Cre excision removes all protein coding sequence. A separate Flpe recombinase is then expressed removes a stop cassette at a eGFP expression cassette in the *ROSA26* locus. (B) Following Cre-mediated excision of *Foxg1*, ~70% of cells are GFP-positive. The proportion of GFP cells remains constant over multiple passages. (C) ICC of *Foxg1*$^{-/-}$ and parental control (*Foxg1*) cells in EGF/FGF-2 or BMP4, for the NS cell markers Nestin and Olig2 and the

astrocyte marker Gfap, respectively. (D) Schematic representations of domains in the FOXG1 and SOX2 proteins. Overall protein sequence homology of mouse Foxg1 to human FOXG1 is 97%. The annotated forkhead binding domain, groucho binding domain (GBD) and jarid binding domain (JBD) demonstrate 100% protein homology between mouse and human. Overall protein sequence homology of mouse Sox2 to human SOX2 is also 97%. The HMG binding domain protein sequence is 100% homologous between mouse and human, and the transactivation domains are identical save for serine-295 which is substituted for alanine in the mouse. (E) and (F) Schematics of Tet-on V5 epitope-tagged FOXG1 (E) or SOX2 (F) transgene cassettes used to establish clonal ANS4 derivatives F6 and S15 by piggyBac recombination. (TRE, TET-responsive element; V5, V5 epitope tag; PB, piggyBac; BSD, blasticidin resistance; IRES, internal ribosome entry site).

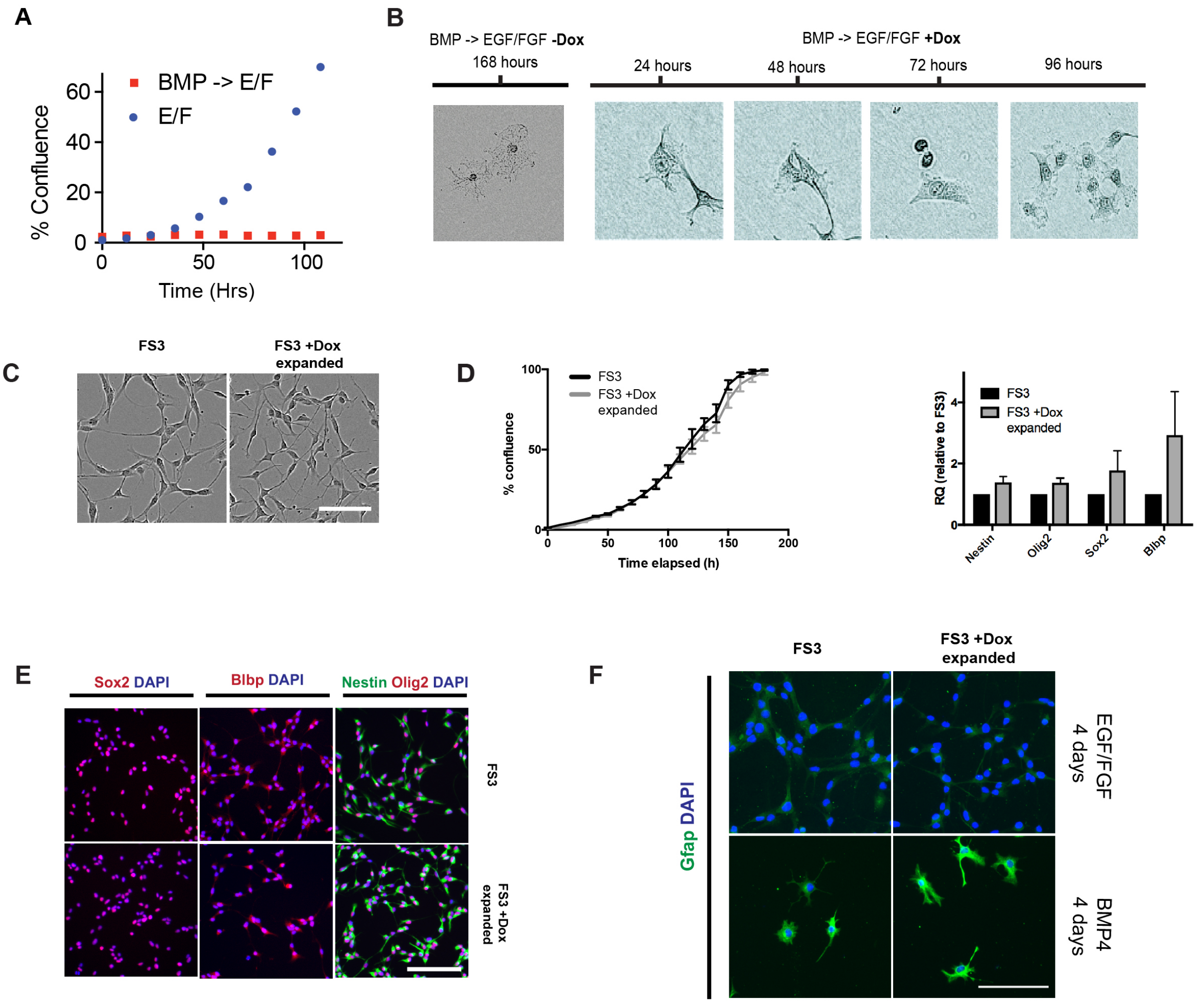

Figure S3 | (A) Incucyte-generated growth curve for FS3 cells plated at 10 cells/mm^2, either in EGF/FGF-2 or treated with BMP4 for 24 h then returned to self-renewal medium. (B) Time-lapse video stills (from Supplementary Video 1) demonstrate colony initiation of BMP4-treated cells with a first division 24–48 hours after re-exposure to EGF/FGF-2 and transgene induction by Dox. Subsequent divisions occur every ~24 h as for parental NS cells. Without Dox addition, cell division does not occur, as demonstrated in first panel. (C) Following 24 h BMP treatment and 14 days of Dox-induced transgene expression in EGF/FGF-2, the

resulting dedifferentiated cells were expanded without Dox. These cells (FS3 +Dox expanded) displayed characteristic NS cell morphology, as shown by phase-contrast images alongside FS3 Scale bar: 100 μm. (D) 'FS3 +Dox expanded' cells self-renew in growth factors with kinetics similar to parental FS3 cells. qRT-PCR analysis (right panel) of the NS cell markers Nestin, Olig2 and Sox2 and the radial glia marker, Blbp, show that the de-differentiated cells express these markers to the same or higher levels than parental FS3. Mean +/- SD, n=3. (E) ICC for Nestin, Olig2, Sox2 and Blbp shows uniform expression in both FS3 and 'FS3 +Dox expanded' cells. Scale bar: 100 μm (F) ICC for Gfap shows both FS3 and de-differentiated (FS3 +Dox expanded) cells display astrocytic differentiation after 4 days treatment with BMP4 (10 ng/ml). Scale bar: 100 μm.

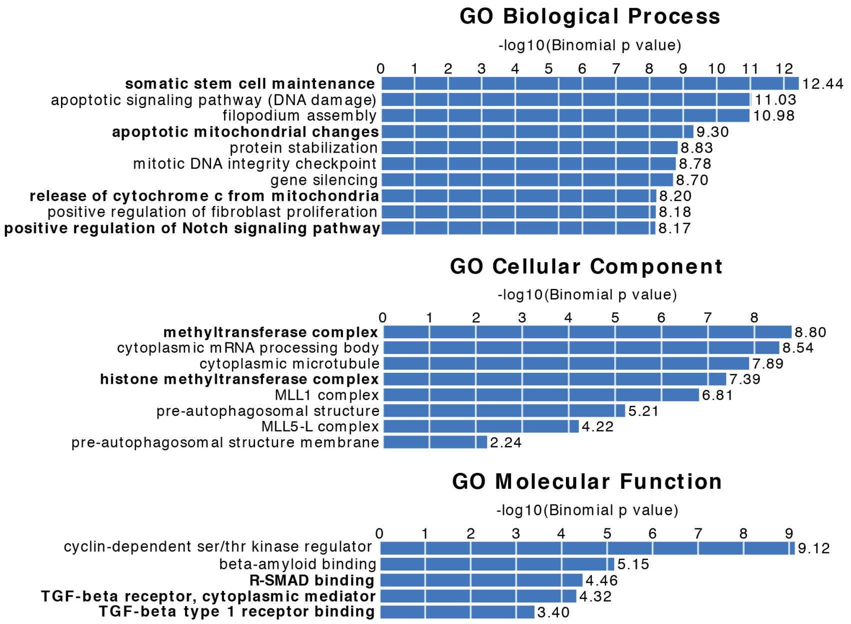

Figure S4 | Statistical enrichment of Gene Ontology (GO) terms for genes bound by FOXG1V5. These include stem cell maintenance, mitochondrial function, Notch and TGF-beta/SMAD signalling, and methyltransferase and histone methyltransferase activity (highlighted in bold).

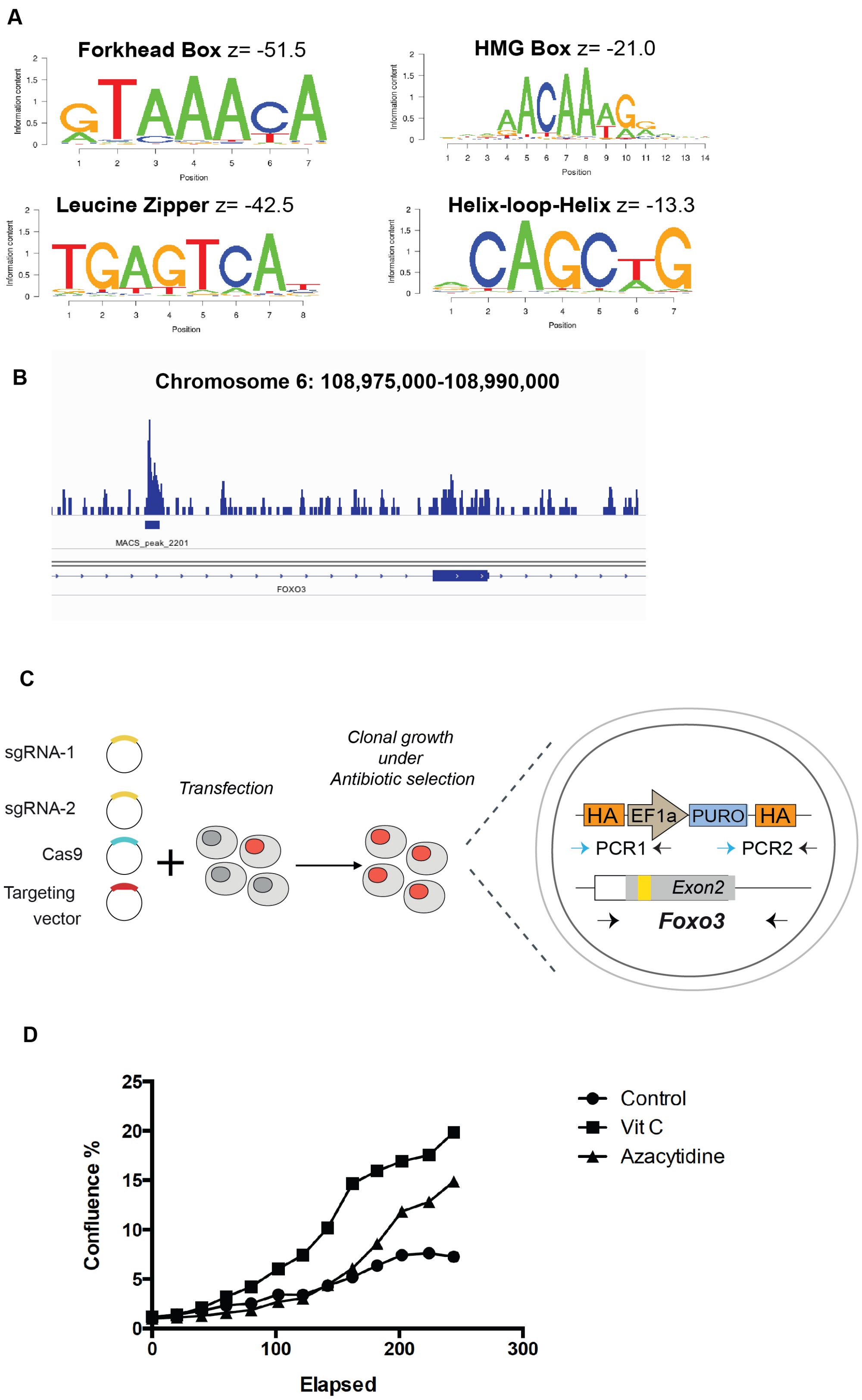

Figure S5 | (A) FOXG1 ChIP-seq using an antibody against native human protein in 4 human GNS lines revealed 7499 peaks, with similar motif enrichments to mouse FOXG1-V5 ChIP-seq. (B) Human native antibody ChIP-seq demonstrates a FOXG1-

bound CIE at the *FOXO3* locus. (C) Schematic depicting the strategy used to delete the critical coding exon 2 of *Foxo3* using CRISPR/Cas9-assisted gene targeting. (D) Growth curves of *Foxo3* mutant cells in the presence of either 5-azacytidine or ascorbic acid (vitamin C).

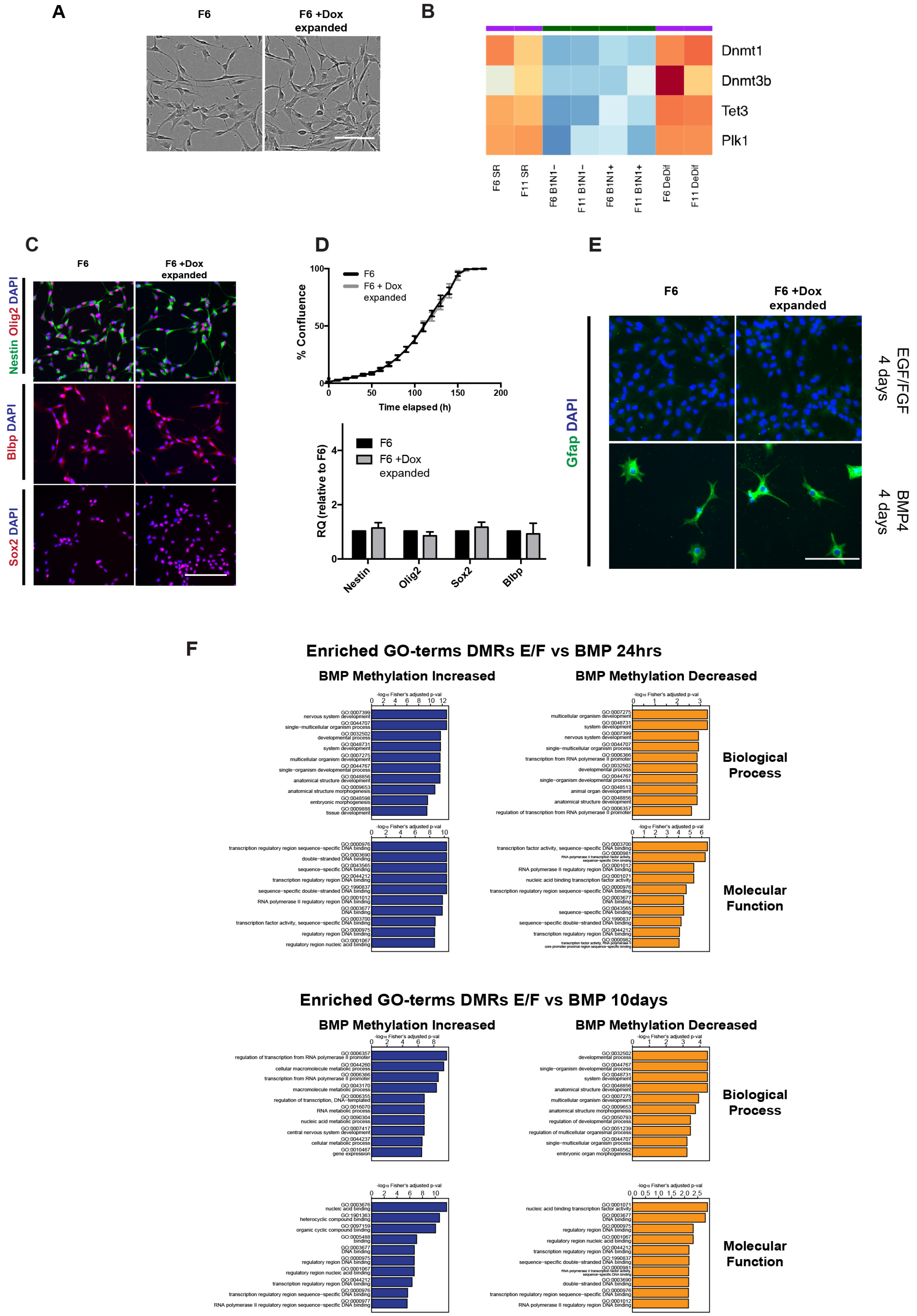
A
F6
F6 +Dox expanded
B
Dnmt1
Dnmt3b
Tet3
Plk1
F6 SR
F11 SR
F6 B1N1−
F11 B1N1−
F6 B1N1+
F11 B1N1+
F6 DeDif
F11 DeDif
C
F6
F6 +Dox expanded
Nestin Olig2 DAPI
Blbp DAPI
Sox2 DAPI
D
% Confluence
Time elapsed (h)
F6
F6 + Dox expanded
RQ (relative to F6)
Nestin
Olig2
Sox2
Blbp
E
F6
F6 +Dox expanded
Gfap DAPI
EGF/FGF 4 days
BMP4 4 days
F
Enriched GO-terms DMRs E/F vs BMP 24hrs
BMP Methylation Increased
BMP Methylation Decreased
Biological Process
Molecular Function
Enriched GO-terms DMRs E/F vs BMP 10days
BMP Methylation Increased
BMP Methylation Decreased
Biological Process
Molecular Function

Figure S6 | (A) Following BMP treatment for 24 h and 14 days of Dox-induced transgene expression in EGF/FGF-2, dedifferentiated cells were expanded without Dox. These cells (F6 +Dox expanded) displayed characteristic NS cell morphology, as shown by phase-contrast images alongside F6. Scale bar: 100 µm. (B) Differentially expressed genes in F6 cells, derived by RNA-seq analysis across the conditions shown in biological duplicate. Following BMP treatment, FOXG1 induction leads to reinstatement over 14 days of an NS-like trancriptome correlating with the NS-like colonies observed. B1N1: BMP-4 treatment for 1 day followed by return to NS cell medium for 1 day. SR: self-renewal. (C) ICC for Nestin, Olig2, Sox2 and Blbp shows uniform expression in both F6 and 'F6 +Dox expanded' cells. Scale bar: 100 µm. (D) 'F6 +Dox expanded' cells self-renew in growth factors with kinetics similar to parental F6 cells. qRT-PCR analysis (below) of the NS cell markers Nestin, Olig2 and Sox2 and the radial glia marker Blbp show that de-differentiated cells express these markers to the same or higher levels than parental F6. Mean +/- SD, n=3. (E) ICC for Gfap shows both F6 and de-differentiated (F6 +Dox expanded) cells display astrocytic differentiation after 4 days of treatment with BMP4 (10 ng/ml). Scale bar: 100 µm. (F) Analysis of GO terms associated with genes located in the vicinity of DMRs identified by RRBS. Shown are the top 10 significantly enriched Biological Process and Molecular Function GO terms associated for BMP-induced DMRs after either 24 h or 10 days in differentiation conditions. Log_{10} Benjami-Hochberg adjusted p-values from Fisher's exact tests versus the background set are plotted.

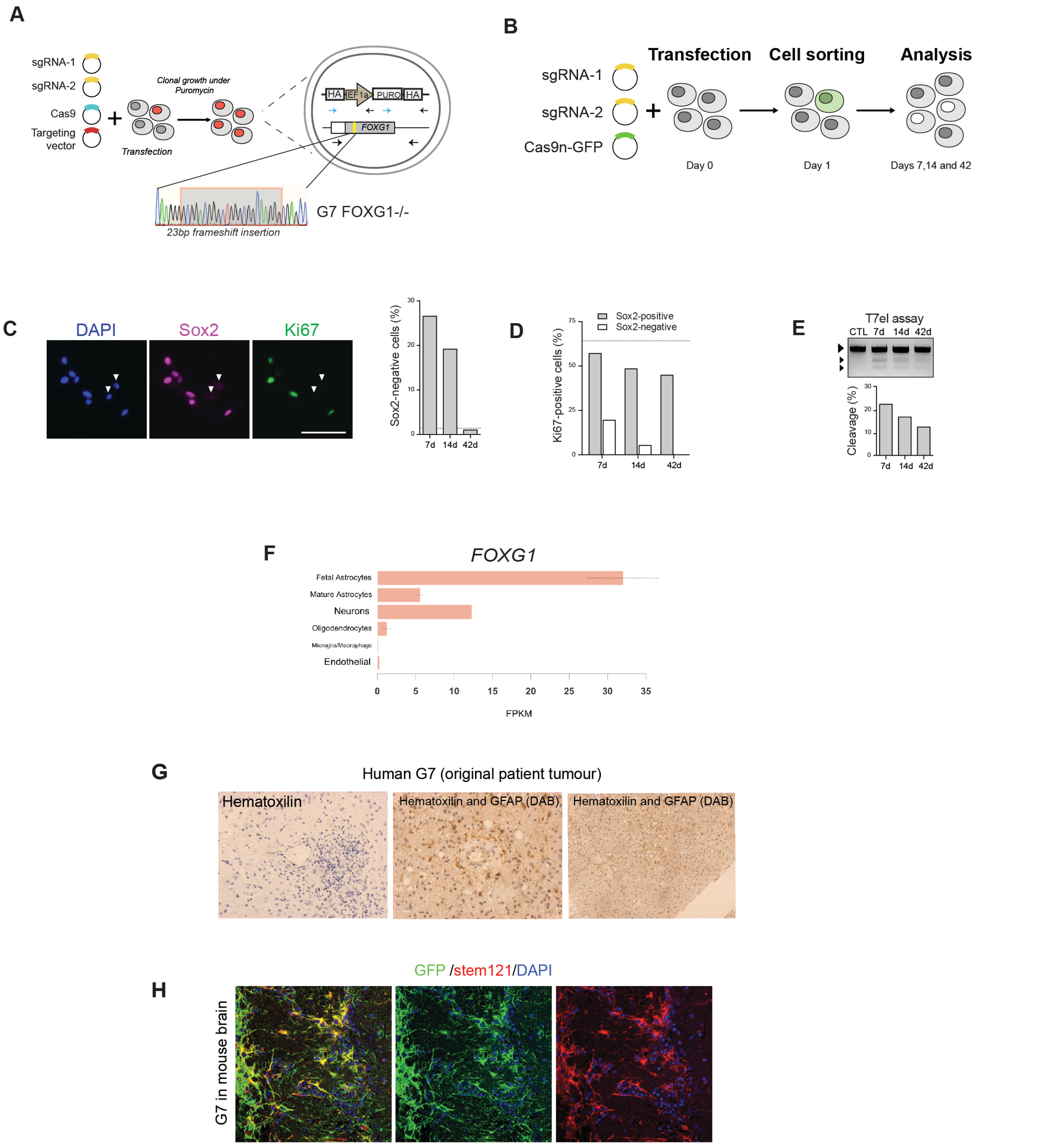

Figure S7 | (A) Schematic of the gene targeting strategy used to eliminate *FOXG1* in G7 cells. Sequencing of the non-targeted allele, which has no detectable protein expression as shown in Figure 7A, demonstrated a 23 bp base frameshift insertion in this clone, consistent with a biallelic knockout. (B) Experimental strategy for *Sox2*

knockout via CRISPR/Cas9-induced NHEJ in GNS cell line G7. Cells were transiently transfected with the CRISPR sgRNA pair together with a Cas9n-2A-GFP plasmid. Control cells were transfected with the Cas-2A-GFP only. Following FACS-based enrichment, transfected cells were subjected to immunocytochemistry analysis and T7eI assay at days 7, 14 and 42 post-transfection. (C) Immunocytochemistry for Sox2 and Ki67 was used to determine the frequency of biallelic knockout. (D) Proliferation rate of *Sox2*-deleted cells at the different time points. Dashed lines represent the Cas9 control values. Scale bar: 100um. (E) Generation of indel mutations in sorted cells at different time points was assessed using a T7EI cleavage assay. Larger arrow indicates the predicted uncleaved PCR product; smaller indicate T7EI-cleaved fragments used to estimate indel frequency. (F) RNA-seq data from neural cell types obtained from the Barres lab database (http://web.stanford.edu/group/barres_lab/brain_rnaseq.html) confirms that FOXG1 is expressed highly in human foetal astrocytes but at lower levels in post-mitotic adult astrocytes. (G) Human G7 histopathology from original patient biopsy. GFAP staining showing abundant microvasculature and GFAP-positive cells. (H) Human G7 cells following xenotransplantion. Stem121 antibody (red) is a human-specific antibody that marks human cytoplasm and confirms engraftment of cells.

A

Cell line	Description	BMP	BMP->EGF/FGF	BMP->E/F/Dox	BMP->E/F/5-aza
IENS	Mouse NS Ink4Arf-/-; EGFRvIII Over	Stable cycle exit	Colony formation/ Dedifferentiation	Not analysed	Not analysed
ANS4	Mouse NS	Stable cycle exit	Stable cycle exit	Stable cycle exit	Stable cycle exit
F6	ANS4 Tet-On FOXG1V5	Stable cycle exit	Stable cycle exit	Colony formation/ Dedifferentiation	Stable cycle exit
S15	ANS4 Tet-On SOX2	Stable cycle exit	Stable cycle exit	Stable cycle exit	Stable cycle exit
FS3	ANS4 Tet-On FOXG1V5-P2A-SOX2	Stable cycle exit	Stable cycle exit	Colony formation/ Dedifferentiation	Stable cycle exit
FOD3	FS3 Foxo3-/-	Stable cycle exit	Stable cycle exit	Colony formation/ Dedifferentiation	Colony formation/ Dedifferentiation
FID11	FS3 Foxo3 cis regulatory region -/-	Stable cycle exit	Stable cycle exit	Stable cycle exit	Stable cycle exit

(red)	Stable cycle exit (* = slow cycle with retained astrocyte markers)
(green)	Colony formation/ Dedifferentiation
(grey)	Not analysed

B

FOXG1 Targeting

FOXG1 5' Arm Forward	aacgacggccagtgaattcgat**aacgaaaatatttccagggt**
FOXG1 5' Arm Reverse	tatcgttatgcgccttgat**cactgagcgctccggcagca**
FOXG1 3' Arm Forward	ctgagctagccatcagtgat**gaagaagaacggcaagtacg**
FOXG1 3' Arm Forward	ccatgattacgccaagcttgat**ccaagtgcattttctagaac**
Guide RNA 1	*ccg*ccctggacggggctaa
Guide RNA 2	gcaagggcgagccgggc*gg*

Foxo3 Targeting

FoxO3 5' Fwd	aacgacggccagtgaattcgat**actctgtggaaggaagttgc**
FoxO3 5' Rev	tatcgttatgcgccttgat**aggaaggtccagttcaaaca**
FoxO3 3' Fwd	ctgagctagccatcagtgat**actctttatgtcctctgttg**
FoxO3 3' Rev	ccatgattacgccaagcttgat**cccttggtacgtccgacctg**
FoxO3 gRNA 1	cgcgttcagaatgaaggcac*ggg*
FoxO3 gRNA 2	cgcatgaagcggctgtgca*ggg*

Foxo3 Cis Regulatory Region Deletion

FoxO3 ID gRNA A	gtaacacaacagaggatgaa*agg*
FoxO3 ID gRNA B	gaaagagcttcttgtggacc*agg*

Supplementary Table 1 | Summary of the mouse cell lines derived and characterised in this study, with response to BMP treatment and return to growth factors as indicated. (B) The guide RNA sequences and targeting vector primers employed for CRISPR/Cas mediated deletion and targeting are listed.

Supplementary Table 2 | Gene ontology analysis of FOXG1 and SOX2 bound genes identified by ChIP-seq.

Supplementary Video 1. Related to Figure 2.

Time-lapse imaging of FS3 cells plated at clonal density treated with BMP then exposed to growth factors + Dox (1 second = 12 hours). A single cell is seen to re-enter cycle at the 48 h time point, then begin dividing with a cycle time of ~24 h.

Supplemental experimental procedures

Guide RNA design and cloning

Guide RNA sequences were selected using the Zifit tool (zifit.partners.org/ZiFiT) to bind at instances of the PAM sequence occurring within gene open reading frames, with low predicted off-target binding.

Oligonucleotides were annealed to produce double-stranded guide inserts, with 4-base overhangs for ligation into a U6 expression plasmid backbone (gift from S. Gerety, Sanger Institute). The guide inserts were phosphorylated, and the U6 vector (SP 117) digested with Bsa1 to generate matching overhangs. Backbone and insert were ligated with T4 DNA ligase (Fermentas) according to manufacturers' guidelines. The resulting plasmids were checked by restriction digest (Nhe1/EcoR1) to confirm incorporation of an insert and then verified by DNA sequencing.

Gene targeting and inducible vector construction

TetON 3G inducible expression cassettes were delivered via piggyBac transposition (Guo et al., 2009). FOXG1 and SOX2 open reading frame sequences were amplified from OriGene plasmid library stocks using PCR primers incorporating Gateway-compatible flanking sequences. The FOXG1-2A-SOX2 cassette was constructed by Gibson assembly (Gibson et al., 2009).

For Cas9-mediated gene targeting, homology arms comprising ~1 kb of genomic sequence flanking the target exon were generated by Phusion PCR from genomic DNA template. These included overhangs complementary to a targeting vector backbone (gift of W. Skarnes, Sanger Institute). The intermediate targeting vector was produced using a 4-part Gibson assembly comprising these arms and a double-

digested intermediate targeting vector backbone. Gateway recombination was then applied to insert an EF1a-PURO selection cassette to produce the final targeting vector.

PCR-based genotyping of targeted clones

For genomic DNA isolation, each well of a confluent 24-well plate was treated with 40 µl lysis buffer (0.45% NP40, 0.45% Tween20, 1x NEB LongAmp PCR buffer) containing 0.2 mg ml^{-1} proteinase K (Sigma). After a 2 h digestion at 55 °C, samples were heated to 95 °C (10 min) and 1–2 µl of the lysate was used in a 10 µl PCR reaction. PCR mix consisted of 0.2 µl DMSO (100% v/v, Sigma), 0.3 µl dNTPs (10 mM, Thermo Fisher Scientific), 2.0 µl 5x LongAMP buffer (NEB), 0.4 µl LongAMP Taq DNA polymerase (NEB), and 12 pmol of each primer. Thermocycler parameters were as follows: 1 cycle 94 °C for 3 min; 40 cycles 94 °C for 15 s, 60 °C for 30 s, 65 °C for 2 min; followed by final extension at 65 °C for 10 min.

For each targeted locus, two sets of genotyping primers spanning the junction of genomic sequences and targeting vector were used. Gene-specific primers outside each end of the 5′ and 3′ homology arms were used in combination with the appropriate universal cassette primers (either CAG-Blasticidin for targeting *Rosa26* and *AAVS1* loci, or Ef1α -Puro for knockout experiments). To identify NHEJ-based damage on the second, non-targeted alleles, regions flanking the sgRNA target sites (500–600 bp) were amplified using gene-specific primers and assessed by DNA sequencing (Source Bioscience).

RNA-seq data analysis

Reads were aligned to the mouse reference genome (mm10/GRCm38) augmented with the Foxg1 expression plasmid sequence with STAR 2.5.2a (Dobin et al., 2013) using the two-pass method for novel splice detection (Engström et al., 2013). Cuffnorm (Trapnell et al., 2012) was used to extract FPKM type expression values for non-mitochondrial protein-coding genes. Intersection of FOXG1 and SOX2 ChIP-seq binding sites with gene promotors was considered if a ChIP peak overlapped a region 1000bp upstream and 200bp downstream of the TSS as annotated with PeakAnalyzer (Salmon-Divon et al., 2010). Heatmaps display genes with high expression level fold changes (adj. $p < 0.01$).

Reduced Representation Bisulfite Sequencing data analysis

Adaptors were removed using Trim Galore (v0.4.1, adaptors: AGATCGGAAGAGC and AAATCAAAAAAAC). Diversity bases introduced by the NuGEN Ovation kit to facilite sequencing were then trimmed using a python script provided by NuGEN as detailed in the ovation kit manual. Paired-end alignment to the mouse reference genome (mm10/GRCm38) was then performed using Bismark (v0.16.3, using Bowtie v2.2.6 and parameters: -N 0 -L 20). Read alignments were parsed to quantify methylation levels at CpGs using Bismark (parameters: -p --no_overlap) and Bedgraph conversion modules. Custom AWK and Python scripts were then used to combine data from both strands for each CpG and across samples. This generated a report for each observed CpG with read coverage and frequency of observed methylation status. In all cases conversion efficiency as assessed from reads mapping to λ phage was > 98%.

CpGs were tested for differential methylation between NS cells cultured in EGF/FGF versus those treated with BMP for 24 h or 10 days. CpGs with coverage < 10 in any sample analysed in each comparison were excluded from the analysis. CpGs were tested for differential methylation using Fisher's exact tests of total and methylated coverage based on running sums of 5 CpGs. Only those CpGs with $p < 0.05$ and changing in the same direction in all 3 replicates were considered significant. DMRs were then defined from the analysis of 5-CpG windows containing at least 3 significant CpGs where the inter-CpG distance did not exceed 200 bp.

CpGs located in significant differentially methylated regions (DMRs) were associated with the most proximal TSS using ChIPpeakAnno (Zhu et al., 2010) and biomaRt (Durinck et al., 2005) against annotation from Ensembl 87. These were compared to polycomb-marked genes, as profiled for H3K27me3 and H3K27me4+H3K27me3 in mouse ES cells, NS cells and brain (Meissner et al., 2008). Enriched loci were compared to a background of all CpGs located in clusters of 5 CpGs were the inter-CpG distance did not exceed 200 bp (i.e., those that could potentially be included in DMRs). Significance was assessed using Fisher's exact tests. GO terms were checked for enrichment at DMR-associated genes using the Bioconductor packages biomaRt and GO.db to annotate genes to DMRs, and Benjami-Hochberg corrected Fisher's exact tests for enrichment versus the background set of CpGs as above.

References

Dobin A, Davis CA, Schlesinger F, Drenkow J, Zaleski C, Jha S, Batut P, Chaisson M, Gingeras TR. 2013. STAR: ultrafast universal RNA-seq aligner. *Bioinformatics* 29: 15–21.

Durinck S, Moreau Y, Kasprzyk A, Davis S, De Moor B, Brazma A, Huber W. 2005. BioMart and Bioconductor: a powerful link between biological databases and microarray data analysis. *Bioinformatics* 21: 3439–40.

Engström PG, Steijger T, Sipos B, Grant GR, Kahles A, Rätsch G, Goldman N, Hubbard TJ, Harrow J, Guigó R, et al. 2013. Systematic evaluation of spliced alignment programs for RNA-seq data. *Nat Meth* 10: 1185–1191.

Gibson DG. 2009. Synthesis of DNA fragments in yeast by one-step assembly of overlapping oligonucleotides. *Nucleic Acids Res* 37: 6984–6990.

Guo G, Yang J, Nichols J, Hall JS, Eyres I, Mansfield W, Smith A. 2009. Klf4 reverts developmentally programmed restriction of ground state pluripotency. *Development* 136: 1063–1069.

Meissner A, Mikkelsen TS, Gu H, Wernig M, Hanna J, Sivachenko A, Zhang X, Bernstein BE, Nusbaum C, Jaffe DB, et al. 2008. Genome-scale DNA methylation maps of pluripotent and differentiated cells. *Nature* 454: 766–70.

Salmon-Divon M, Dvinge H, Tammoja K, Bertone P. 2010. PeakAnalyzer: genome-wide annotation of chromatin binding and modification loci. *BMC Bioinformatics* 11: 415.

Trapnell C, Roberts A, Goff L, Pertea G, Kim D, Kelley DR, Pimentel H, Salzberg SL, Rinn JL, Pachter L. 2012. Differential gene and transcript expression analysis of RNA-seq experiments with TopHat and Cufflinks. *Nat Protoc* 7: 562–578.

Zhu LJ, Gazin C, Lawson ND, Pagès H, Lin SM, Lapointe DS, Green MR. 2010. ChIPpeakAnno: a Bioconductor package to annotate ChIP-seq and ChIP-chip data. *BMC Bioinformatics* 11: 237.
